# The Interplay between Gut Microbiota and Parkinson’s Disease: Implications on Diagnosis and Treatment

**DOI:** 10.3390/ijms232012289

**Published:** 2022-10-14

**Authors:** Angelica Varesi, Lucrezia Irene Maria Campagnoli, Foroogh Fahmideh, Elisa Pierella, Marcello Romeo, Giovanni Ricevuti, Marchesi Nicoletta, Salvatore Chirumbolo, Alessia Pascale

**Affiliations:** 1 Department of Biology and Biotechnology, University of Pavia, 27100 Pavia, Italy; 2 Almo Collegio Borromeo, 27100 Pavia, Italy; 3 Department of Drug Sciences, Section of Pharmacology, University of Pavia, 27100 Pavia, Italy; 4 School of Medicine, Faculty of Clinical and Biomedical Sciences, University of Central Lancashire, Preston PR1 2HE, UK; 5 Department of Drug Sciences, University of Pavia, 27100 Pavia, Italy; 6 Department of Neurosciences, Biomedicine and Movement Sciences, University of Verona, 37129 Verona, Italy

**Keywords:** Parkinson’s disease, gut microbiota, dysbiosis, intestinal permeability, diagnosis, probiotics, prebiotics, fecal microbiota transplantation, diet, antibiotics

## Abstract

The bidirectional interaction between the gut microbiota (GM) and the Central Nervous System, the so-called gut microbiota brain axis (GMBA), deeply affects brain function and has an important impact on the development of neurodegenerative diseases. In Parkinson’s disease (PD), gastrointestinal symptoms often precede the onset of motor and non-motor manifestations, and alterations in the GM composition accompany disease pathogenesis. Several studies have been conducted to unravel the role of dysbiosis and intestinal permeability in PD onset and progression, but the therapeutic and diagnostic applications of GM modifying approaches remain to be fully elucidated. After a brief introduction on the involvement of GMBA in the disease, we present evidence for GM alterations and leaky gut in PD patients. According to these data, we then review the potential of GM-based signatures to serve as disease biomarkers and we highlight the emerging role of probiotics, prebiotics, antibiotics, dietary interventions, and fecal microbiota transplantation as supportive therapeutic approaches in PD. Finally, we analyze the mutual influence between commonly prescribed PD medications and gut-microbiota, and we offer insights on the involvement also of nasal and oral microbiota in PD pathology, thus providing a comprehensive and up-to-date overview on the role of microbial features in disease diagnosis and treatment.

## 1. Introduction

The chronic neurodegenerative pathology known as Parkinson’s disease (PD) can be described as an accumulation of a misfolded type of α-synuclein (the so-called Lewy bodies), an event occurring in dopaminergic neurons of the *substantia nigra* (SN), alongside with other related neuronal circuitries, which finally contribute both to non-motor (cognitive disorders, dysfunction in the olfactive sense, complications in the urogenital apparatus and in the gastrointestinal (GI) function) and mainly to motor symptoms (tremors, bradykinesia, abnormal gait and stiffness) [1,2,3,4]. The recent research suggests a relationship between gut microbiota (GM) and PD, due to the close interplay between the GM and the brain, known as “gut microbiota brain axis” (GMBA) [5], as recently reviewed in [6,7]. GMBA is usually described as a bidirectional functional system linking the enteric nervous system (ENS) in the gastrointestinal tract with the brain [5], but within the complex activity of our nervous system it might have a role much more puzzling and complicated than expected so far. The interplay of the GMBA with the immune system is particularly crucial [8,9]. A first, perhaps naïve, consideration is that a healthy composition of the GM warrants the integrity not only of the intestinal immune barrier, but also of the blood-brain barrier (BBB), via the regulation of the expression of fundamental proteins in the BBB such as tight junctions (i.e., claudin-5 and occludin), using the bacteria-produced short chain fatty acids (SCFAs) as modulatory signals for the brain synaptogenesis and development [10,11]. SCFAs are anti-inflammatory molecules with a key role in the GMBA [12] and some evidence reported that SCFAs-producing bacteria are reduced in PD [13]. The relationship between PD and GMBA has been also supported by the observation that α-synuclein is produced by the enteric neurons in the ENS, probably with functions related to uptake and neurotransmission [14]. Further, recent evidence has shown that a gut bacterial amyloid promotes the aggregation of α-synuclein, causing motor impairments in experimental mice [15]. The review by Mulak et al. addresses the issue of PD and GMBA as a particular item in the widest topic on the interplay between GM and brain in neurodegenerative disorders and indicated at least four levels of action [16]. The first level is represented by ENS, with neurons of sub-mucosal (Meissner’s) and myenteric (Auerbach’s) plexi, alongside the enteric glial cells [17]. The second level is represented by paravertebral ganglia, which modulate several visceral reflex responses at a peripheral level [18]. The third level is represented by the autonomous nervous system, while the fourth one is represented by the Central Nervous System (CNS) [16].

However, it seems that the current, more widely spread hypothesis about the etiopathogenetic role of GM in PD, is led by the ENS-derived α-synuclein (i.e., gut bacteria may induce the aggregation of α-synuclein in ENS), which, propagating in a prion-like manner, passes to the brain through the vagus nerve [15,19,20]. Notably, *Desulfovibrio* bacteria have been strictly associated with PD [21], where specifically hydrogen sulfide, which is also a known neuromodulator [22] produced by gut bacteria (*Desulfovibrionaceae* and *Enterobacteriaceae* families), has been associated with the etiopathogenesis of the disease [23].

The role of GMBA in PD may be considered, for certain aspects, a novel intriguing chapter in the complexity characterizing PD pathogenesis and evolution [24,25,26], for which the role of immunity (particularly T-reg cells) might be fundamental [27]. Nevertheless, further insights are needed to better disclose this captivating relationship.

## 2. Parkinson’s Disease and Gut Microbiota: Links and Mechanisms

### 2.1. Gut Microbiota Dysregulation in PD

**Figure 1:**
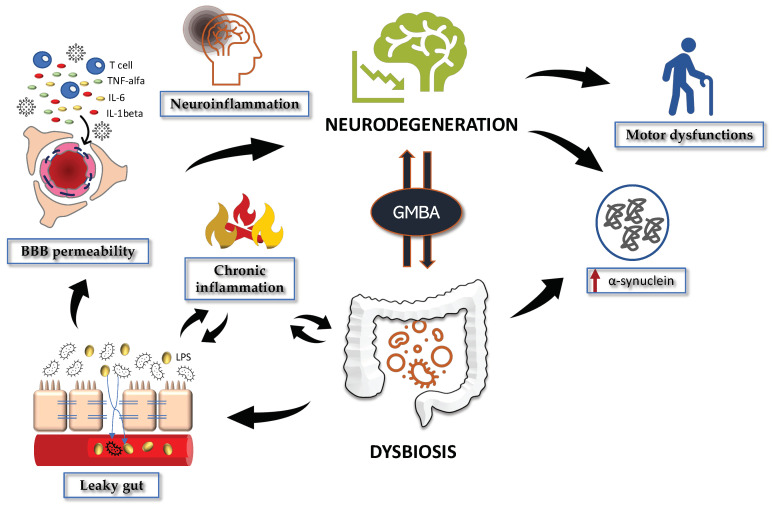
The gut microbiota brain axis (GMBA) in Parkinson’s disease (PD). In PD, α-synuclein aggregates are retrieved both in the dopaminergic neurons of the *substantia nigra*, as well as in the gut, and often associate to intestinal dysbiosis. Dysregulations in the gut microbiota composition trigger chronic inflammation and blood brain barrier (BBB) disruption through increased gut permeability (the so-called leaky gut). The resulting neuroinflammation then directly or indirectly promotes cognitive decline and motor dysfunction, which are the typical PD manifestations. ↑: increase.

Gut dysbiosis is defined as an imbalance of the intestinal flora due to an overgrowth of harmful taxa at the expense of beneficial commensal bacteria [28]. To date, this condition is known to participate in the pathophysiology of several GI and extraintestinal disorders, such as intestinal bowel syndrome, diabetes, obesity, chronic fatigue syndrome, autoimmune diseases, and several neuropsychiatric and neurologic disorders, including neurodegeneration [28,29,30,31,32,33,34,35,36,37,38,39]. Concerning PD, dysbiosis followed by GI symptoms and gut discomfort far precedes the onset of motor dysfunctions and it is linked to neuroinflammation, as well as to alterations in dopamine, serotonin, and kynurenine metabolism through the gut-brain axis [40,41,42,43,44,45,46] (Figure 1). Similar to what has been reported in Alzheimer’s disease (AD) and aging [30], feces from PD patients are enriched in opportunistic pathogens and pro-inflammatory taxa at the expense of anti-inflammatory microbes and SCFAs-producing bacteria, especially butyrate [47,48,49,50,51]. Accordingly, meta-analysis and systematic reviews report that PD patients are characterized by an overgrowth of the genera *Bifidobacterium*, *Lactobacillus*, *Akkermansia* and of the opportunistic pathogens *Porphyromonas* and *Corynebacterium*, together with a decreased abundance of the SCFAs producers *Prevotellaceae*, *Lachnospiraceae* and *Faecalibacterium* [52,53,54,55,56]. There is evidence that *Faecalibacterium* preserves the gut-barrier function through the production of the SCFA butyrate and the secretion of anti-inflammatory mediators [57,58]. Of note, lower levels of butyrate are linked to postural instability, gait disorders, Movement Disorder Society-Sponsored Revision of the Unified Parkinson’s Disease Rating Scale (MDS-UPDRS) part III motor scores and depression among PD patients showing a reduced count of *Faecalibacterium* [59,60,61,62,63]. Other butyrate-producing taxa, such as *Blautia*, *Caprococcus*, *Rosburia* and *Prevotella*, also exert immunomodulatory activities and are reduced in PD patients compared to controls [50,60,64,65,66]. In line with these data, a study conducted in central China on 39 PD patients and their healthy spouses reported an inverse association between *Prevotella* abundance and disease severity, thus confirming the important role of inflammation in disease progression [64]. 

*Lachnospiraceae* are another family of bacteria underrepresented in PD-related gut microbiomes that contribute to the maintenance of intestinal homeostasis and prevent gut inflammation through the secretion of butyrate [67,68,69]. Their reduced presence, especially that of *Ruminococcus*, has been associated with cognitive impairment as measured by the Mini Mental State Examination (MMSE) test and postural instability in PD patients [59,68,69,70]. A similar phenotype was also associated to an increase in *Lactobacillaceae*, *Enterobacteriaceae* and *Christensenellaceae*, as reported by different studies [69,71,72]. Among *Enterobacteriaceae*, while *Escherichia/Shigella* have been negatively linked with disease duration in a case-control study involving 45 PD patients and their healthy spouses [73], *Klebsiella* showed a positive correlation in an independent report [64]. Concerning motor symptoms, it has been observed that the pro-inflammatory taxa *Escherichia* and *Serratia* are prevalent in non-tremor dominant patients [74], which present a more severe and faster progression of the disease compared to tremor dominant ones [75,76,77]. 

*Akkermansia muciniphila* is a bacterial genus belonging to the family of *Verrucomicrobiaceae* that is frequently found increased in patients with PD compared to controls [65,69,78,79,80,81]. Although *Akkermansia muciniphila* possesses the beneficial ability to convert mucin into SCFAs [82], its mucin degrading activity might also damage the gut-barrier triggering inflammation and promoting gut permeability [83,84]. Therefore, a high abundance of *Akkermansia muciniphila* may accelerate disease progression and favor α-synuclein (the already mentioned crucial protein involved in PD pathology) aggregation in gut enteroendocrine cells [85]. Phenotypically, studies show that increased levels of *Akkermansia muciniphila* are linked to stool consistency and constipation [86,87], but this association has yet to be validated in the context of PD [80].

*Lactobacillus* and *Bifidobacterium* are generally considered beneficial and often included in probiotic mixtures [88]. Their counterintuitive enrichment in stools from PD patients, reported in most studies [41,53,54,68,71,72,79] with few exceptions [78,89,90], could represent a compensatory mechanism to restore intestinal homeostasis [41]. 

There is evidence that GM composition changes according to ethnicity and geographic location [91,92]. Concerning PD, studies conducted on a population-specific cohort of patients shows the prominence of some geographic signatures, albeit on a similar background of bacterial dysbiosis [64,65,66,68,71,72,73,78]. For example, feces from Northern German PD patients showed increased abundance in the *Barnesiellaceae* family [72], while the genera *Butyricicoccus* and *Clostridium* XIVb were linked to a cognitive deficit in a Chinese PD cohort [73]. Among Chinese people, *Parasutterella* and *Bilophila wadsworthia* were more abundant in 39 PD patients compared to their healthy spouses [64], while the Northeastern Han population with PD showed reduced *Bacteroides*, as well as increased *Ruminococcaceae* and *Lachnospiraceae* NK4A [65]. These latter results, although in constrast with the previously described studies reporting lower levels of *Lachnospiraceae* and *Ruminococcus* among patients [59,69,81], could be explained as a population specific trait or may be a result of the small sample size. Furthermore, it has been reported that the Australian signature consists of decreased *Colidextribacter*, *Agathobaculum*, *Kineothrix*, *Roseburia* and *Intestinibacter* in favor of enriched *Synergistetes* and *Proteobacteria*, which elicit inflammation [50,66]. Lastly, feces from PD patients coming from various countries are enriched in *Enterococcaceae* [63,71,72], similar to what has been observed in AD [93]. 

GM and host metabolism are strictly interconnected [94,95]. Indeed, because of dysbiosis, lipid, amino acid and energy metabolism are often dysregulated in PD patients [96,97,98,99,100]. Studies show that a decrease in branched chain and aromatic amino acid biosynthesis, carbohydrate fermentation and butyrate synthesis is accompanied by an upregulation in lipopolysaccharides (LPS) production, bacterial type III secretion system biosynthesis (a complex bacterial structure involved in virulence) and proteolytic fermentation, which trigger inflammation and promote the release of harmful metabolites (i.e., phenylacetylglutamine and p-cresol) [50,101,102]. Furthermore, the pro-inflammatory taxa enriched in the gut of PD patients correlate with higher levels of plasma indole-3-propionic acid, hippuric acid (a carboxylic acid found in urine), as well as of the deoxycholic and glycodeoxycholic bile acids, which alter cholesterol metabolism [98,103]. Other dangerous molecules produced by the GM as a result of dysbiosis and associated to neurodegeneration are sphingolipids, trimethylamine N-oxide (TMAO) and the branched-chain fatty acid succinate that is linked to PD severity [61,104]. TMAO is an amine oxide product of the bacterial metabolism that has been implicated in oxidative stress enhancement, BBB disruption, mitochondria dysfunction, brain aging and neurodegeneration [105,106,107]. Often, increased levels of plasma TMAO are accompanied by a decreased production of several SCFAs required for the proper functioning of the ENS [61,104,108]. It has been shown that low levels of acetate, butyrate and above all propionate in feces from PD patients are linked to worse MDS-UPDRS part III scores [62,109], and the same is true for serum propionic acid [110]. Serum butyric acid and capronic acid are also downregulated in PD patients, while heptanoic acid is upregulated [110]. In plasma, Chen et al. report higher levels of acetate, propionate and butyrate in PD patients compared to controls, with plasma butyrate and valerate being inversely correlated with MMSE scores [62]. These conflicting results between stool and plasma could be due to increased intestinal permeability, a characteristic feature of PD patients (see Section 2.2 Gut permeability in PD) and which would facilitate the entry of SCFAs into the circulation [103]. In this respect, more research is needed to better clarify the relationship between blood and fecal levels of these molecules. 

Given their important role, approaches aimed at restoring the optimal SCFAs levels have been proposed, with promising results [111]. These beneficial effects should be ascribed to the immunomodulatory and gut epithelial barrier-strengthening action of SCFAs, which together counteract inflammation [67]. Concerning PD, propionate supplementation was able to promote neurite outgrowth, tyrosine hydroxylase (TH) expression and dopaminergic cell survival in vitro [112]. in vivo, MPTP (1-methyl-4-phenyl-1,2,3,6-tetrahydropyridine; a prodrug of the neurotoxin MPP+)-treated mice receiving propionate show a better performance in the stepping test, cylinder test and whisker test, in line with previous data [109,110]. Other investigations conducted with sodium butyrate have instead yielded conflicting results. Indeed, while sodium butyrate administration attenuated microglial activation and reduced cognitive deficits in preclinical models [113,114], other studies report increased dopaminergic neuronal toxicity, brain and colon inflammation, oxidative stress, and astrocyte activation both in vitro and in vivo [115,116]. Thus, unlike propionate, the contribution of sodium butyrate on neuroinflammation and gut inflammation remains unclear. 

### 2.2. Gut Permeability in PD

The intestinal barrier is composed of a monolayer of epithelial cells that separate the GM present in the lumen from the internal tissues [117]. Under homeostatic conditions, the strict intercellular connections ensured by desmosomes, tight junctions and adherens junctions regulate the selective passage of nutrients, water and electrolytes while preventing the translocation of bacterial toxins, microorganisms and other harmful molecules [118]. However, disease-associated conditions such as dysbiosis, chronic intestinal inflammation and stress can damage the intestinal barrier integrity, allowing gut microbes to enter the circulation [33,119] (Figure 1). This condition, known as intestinal permeability or leaky gut, has been implicated in the pathophysiology of various GI, as well as extraintestinal disorders [120,121,122], including PD [117,123,124,125,126,127,128]. There is evidence that decreased levels of the colonic tight junction proteins occludin and zonula occludens-1 (ZO-1) trigger intestinal inflammation by activating the caspase-1 inflammasome signaling and increasing the levels of the pro-inflammatory interleukin 1 beta (IL-1β) and tumor necrosis factor alpha (TNF-α) [129,130,131]. Accordingly, higher concentrations of the leaky gut markers zonulin and α1-antitrypsin, as well as enhanced abundance of the colonic inflammatory markers calprotectin and lactoferrin, were noticed in feces from PD patients compared to controls [132,133,134]. These changes are often accompanied by variations in the concentration of stool SCFAs, with a reduction in fecal acetic, butyric, and propionic acids observed in PD patients [135] (see Section 2.1 Gut microbiota dysregulation in PD). Of note, MPTP mice treatment with propionate enhances the expression of ZO-1 and occludin via the Akt signaling, thus preserving the gut epithelial barrier integrity [109]. Other approaches useful to measure the extent of intestinal permeability and the degree of gut absorption are the lactulose/mannitol urinary test, the sucrose urinary assessment, the FITC (fluorescein)-dextran permeability test and the transepithelial resistance analysis [136,137]. In this respect, abnormal lactulose/mannitol ratio and sucrose concentration values found in subjects with PD have been associated with tight junction alterations [138,139]. Similar results were then replicated in 6-OHDA (hydroxydopamine)-treated rats (an animal model of PD), in which an increase in FITC-dextran permeability was observed as opposed to a decrease in transepithelial resistance, indicative of an impaired barrier functionality [140]. Of note, damage to the gut epithelial barrier alone without gut inflammation is not sufficient to explain disease severity and progression, as demonstrated in mice that overexpress human α -synuclein overexpressing (ASO mice) treated with dextran sodium sulfate, which is known to damage the mucosal epithelium [141]. These data demonstrate that a coordination of a series of complex, interconnected and related events is required to connect GI dysfunctions with PD symptoms. 

Upon gut barrier breakdown, gram-negative endobacteria and their derived endotoxins, especially LPS, can enter the bloodstream, a condition known as metabolic endotoxemia [123,142]. Increased levels of LPS in the circulation are known to trigger systemic inflammation and to mediate BBB destruction, which allows microbial-derived inflammatory endotoxins to also enter the brain [123,129,142]. Moreover, LPS is known to interact with α-synuclein and to induce its nucleation [143], which will initiate a cascade of molecular events, leading to the formation of protein aggregates. Systemic administration of LPS and TNF-α to wild type adult mice is sufficient to stimulate the microglial expression of monocyte chemoattractant protein-1 (MCP-1), IL-1β, TNF-α and nuclear factor kappa-light-chain-enhancer of activated B cells (NF-κB) and to enhance TH-positive neuronal loss in the SN, thus contributing to the onset of neurodegeneration [144]. Similarly, early motor symptoms are noticed in a mouse model of PD overexpressing the human α synuclein (also called Thy-1-α synuclein mouse) upon dietary intake of LPS [131]. In line with these data, serum from PD patients contains low levels of LPS binding protein [145,146], which are indicative of a chronic systemic exposure to LPS and gram negative endobacteria [147,148,149]. Accordingly, these changes were accompanied by increased positivity to gram-negative staining in the intestinal mucosa and higher levels of plasma TNF-α and interferon-γ [146,150]. Therapeutically, attempts to reduce systemic inflammation have been made by treating PD mice with analogues of the anti-inflammatory molecules cholecystokinin and glucagon-like peptide1. Results are promising, since reductions in tight junction leakage, colonic inflammation, gut α-synuclein aggregates and dopaminergic neural loss have been observed [151]. 

Although the leaky gut is becoming important for understanding PD pathophysiology, some conflicting evidence remains. In this regard, independent studies report no differences in serum IL-6, C-reactive protein, TNF-α, fecal zonulin, colonic ZO-1 and in the gut mucosal integrity marker serum diamine oxidase in samples obtained from PD patients compared to controls [130,134,145], and no changes in intestinal permeability were observed in mice treated with the PD-inducing compound rotenone, a well-known pesticide [152]. However, therapeutic options aimed at restoring dysbiosis and leaky gut, such as probiotics, prebiotics, fecal microbiota transplantation and dietary interventions appear to be promising and deserve further investigation.

## 3. Parkinson’s Disease and Gut Microbiota: Biomarkers and Drug Interactions

### 3.1. Gut Microbiota-Based PD Biomarkers

One important aspect of PD research is to find reliable, accurate, predictive, non-invasive, sensitive, and specific biomarkers for early disease detection and for following its course [153,154,155]. Since, as mentioned, GI symptoms often precede the onset of brain disorders, GM-based biomarkers have recently been considered as promising early and prodromal diagnostic tools in various neurological diseases, such as multiple sclerosis, AD and PD itself [30,156,157]. Concerning PD, it has been proposed that the severity of GI manifestations may be an early prediction of worse cognitive performances [158], and that the intestinal α-synuclein may function as a prodromal disease biomarker [159]. Moreover, GM composition might be used to monitor disease onset and progression [160]. In this respect, metagenomic sequencing, followed by random forest machine learning approaches, turned out to be the best method for new biomarker discovery in terms of precision and accuracy [161]. Accordingly, Qian et al. reported that a combination of 25 genetic microbial markers could effectively distinguish not only cases from controls but also differentially diagnose PD, AD and multiple system atrophy patients [157]. Among the bacterial features, a reduction of *Prevotellaceae* together with a rise in *Akkermansia* have been proposed as a possible PD diagnostic signature [79]. More recently, Guo et al. reported that the simultaneous measurement of *Blautia* levels in the feces, brain and blood can be used to differentiate cases from controls, as PD patients show reduced abundance of this genus [162]. Of note, improvements in predictive ability can be achieved by combining microbiome composition data with dietary information [163].

Besides looking for biomarkers based on the gut microbiota composition, other attempts have been made by measuring the abundance of GM-related molecules and/or SCFAs in the circulation [104]. For example, dementia development may be predicted by low levels of plasma TMAO measured at an early stage of the disease, thus serving as prognostic marker [164]. Moreover, disease severity has been associated with lower urine levels of urolithin, an anti-inflammatory molecule produced by the gut microbiota upon polyphenols intake [100]. When low, urolithin reflects a condition of dysbiosis characterized by an overgrowth of the pro-inflammatory *Enterobacteriaceae* at the expense of the beneficial *Lachnospiraceae* and *Gordonibacter* [100]. Another possible marker, namely blood LPS binding protein, has been shown to differentiate PD from controls without discriminating the disease stage; however the high variability measured among patients currently prevents its clinical application [147]. Concerning SCFAs, a study conducted by He et al. on 25 multiple system atrophy, 46 PD patients and 46 controls showed that lower plasma levels of acetic acid and propionic acid could discriminate the phenotypically similar multiple system atrophy and PD with 80% sensitivity and 91% specificity, thus enabling differential diagnosis among synucleinopathies [165]. 

Predicting and monitoring disease progression is of utmost importance to carry out personalized treatments [155]. So far, few studies have investigated the relationship between the GM and the rate of PD worsening, although with promising results. In 2021, Cilia et al. showed that a shortage of *Roseburia* in de novo PD patients was predictive of a faster deterioration in motor, non-motor, and intellectual ability within 3 years [160]. Similar results were then obtained by Lubomski et al. who reported that low levels of *Barnesiella* at the baseline and at 1-year follow-up were associated with a worse clinical evolution of PD [51]. Furthermore, a machine learning approach accounting for the abundance of various gut microbial species revealed that a reduction in *Fusicatenibacter*, *Blautia* and *Faecalibacterium*, together with an increased presence of *Akkermansia*, correlates with a faster progression of the disease [166]. Of note, patients without worsening symptoms over a one-year period (according to the Haehn&Yahr staging-scale and MPS-UPDRS-PartIII) showed stability in alpha and beta diversity, as well as gut microbiota composition, thus confirming the key role of gut microbiota in disease progression [167].

**Figure 2:**
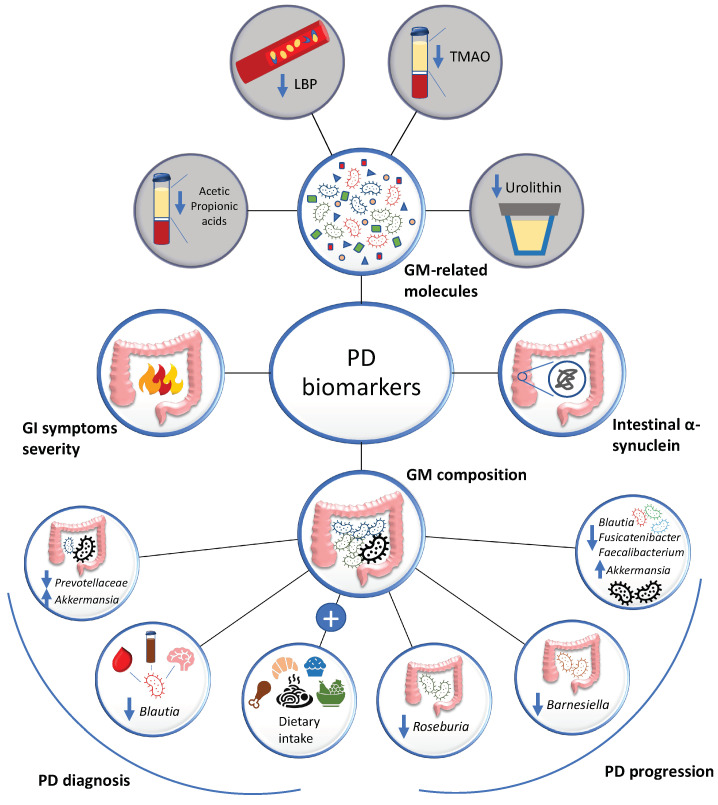
Gut microbiota (GM)-based Parkinson’s disease (PD) biomarkers. Four different traits of the intestine have been proposed as PD biomarkers: intestinal α-synuclein, gastrointestinal (GI) symptoms severity, GM-related molecules, and GM composition. Among GM-related molecules, low levels of urine urolithin, decreased plasma trimethylamine N-oxide (TMAO), reduced plasma acetic and propionic acids and low levels of circulating LPS binding protein (LBP) are associated with PD. Concerning GM composition, while decreased relative abundance of *Roseburia*, *Barnesiella*, *Blautia*, *Fusicatenibacter*, *Faecalibacterium* and increased *Akkermansia* may serve as indicators of PD progression, dysbiosis data alone or associated with dietary intake information are useful for PD diagnosis. ↑: increase; ↓: decrease.

A summary of the main proposed GM-based PD biomarkers is shown in Figure 2. 

Although conflicting evidence still exists [168] and data remain limited, the results of new clinical trials, such as the Dutch Parkinson Cohort (DUPARC) prospective cohort study, will be crucial to better define the possible role of GM as alternative PD biomarker for early diagnosis, differential discrimination, and progression monitoring [169]. Possibly, the use of several multimodal biomarkers in combination may strengthen the predictive capability by improving specificity and sensitivity [153,154].

### 3.2. Gut Microbiota-Drug Interactions

The mutual influence between GM and drug intake is well reported in the literature, and a growing body of evidence is emerging on the relationship between commonly prescribed PD drugs and the GM profile [170,171,172,173,174,175,176,177,178]. 

Levodopa (L-dopa) is a dopamine precursor and the leading compound for the treatment of PD [179]. To prevent its early conversion in dopamine before it reaches the brain, L-dopa is usually taken in combination with a dopa decarboxylase inhibitor, such as carbidopa [176,180]. However, carbidopa is not effective against the bacterial dopa decarboxylases [180], and this enables the GM to metabolize L-dopa, decreasing the drug availability while increasing the side effects [180,181]. Indeed, in the intestine, L-dopa is first transformed into dopamine by a dopa decarboxylase from *E. faecalis* and then converted into m-tyramine through the action of a dehydroxylase from *Eggerthella lenta* [181,182,183]. At the same time, *C. sporogenes* can also deaminate L-dopa, originating the metabolite 3-(3,4-dihydroxyphenyl) propionic acid, which is reported to impair ileal mobility [184]. Of note, considerable levels of this metabolite were reported in feces of L-dopa treated PD patients [184]. Finally, another bacterial species that is found with higher prevalence in PD patients compared to healthy subjects is *Helicobacter pylori.* Interestingly, the binding of L-dopa to *H. pylori* contributes to the reduced absorption of the drug, resulting in motor impairment [29]. 

In addition to being dependent on intestinal bacteria, L-dopa influences the GM composition itself. Accordingly, an increased relative abundance of *Peptoniphilus*, *Finegoldia* and *Enterococcus*, as well as a reduced presence of *Faecalibacterium*, *Blautia* and *Lachnospirae* were reported upon L-dopa exposure [185,186]. Treatment formulation may also change bacterial abundance. In this respect, patients receiving an L-dopa + carbidopa intestinal gel display higher levels of *Enterobacteriaceae*, *Escherichia* and *Serratia* compared to those receiving only L-dopa, while both groups show metabolic markers of gut inflammation [186]. In contrast, results from another study conducted on 19 PD patients before and after a 90-day long treatment with L-dopa reported no major differences in either α or β diversity between the two time points, suggesting that more research is needed to better clarify which factors are implicated in the L-dopa-mediated GM reshaping [187]. 

Catechol-o-methyl transferase (COMT) inhibitors, anticholinergics, monoaminoxidase inhibitors and dopamine agonists are additional PD drugs that are administered with or without L-dopa and may condition the dopaminergic balance [185]. Indeed, there is evidence that these medications have an impact on the abundance of the gut microbial dopa decarboxylases, thus potentially influencing dopamine metabolism [188]. Moreover, their intake has recently also been correlated with gut microbiota reshaping, although conflicting evidence remains [189,190]. For instance, treatment with dopamine agonists was associated with reduced intestinal motility and small intestinal bacteria overgrowth (SIBO; a kind of dysbiosis that frequently occurs in PD patients) in rats [191]. These effects were mediated by a greater relative abundance of *Lactobacillus* and *Bifidobacterium*, coupled with a decrease in *Lachnospiraceae* and *Prevotellaceae* [191]. In addition, COMT inhibitors and anticholinergics are known to induce GI side effects [192,193,194], which may be due to an imbalance of the intestinal flora towards harmful bacteria [190]. Further studies showed that certain gut microbial signatures, such as increased *Bifidobacterium* or *Lactobacillaceae*, are present in PD patients treated with COMT inhibitors [52,195,196,197]. In addition, a substantial decrease in *Faecalibacterium prausnitzii* abundance coupled with a trend towards lower fecal butyrate associate with entacapone, a widely prescribed COMT inhibitor [198], but not with the analogues opicapone and tolcapone (the latter one has been withdrawn from the market for safety reasons) [108,199]. Other studies confirmed the alteration of taxa related to GI disorders and constipation upon entacapone intake [185,200]. In particular, Fu et al. showed reduced *Sellimonas*, *Lactobacillus*, *Faecalibacterium*, *Dorea*, *Intestinobacter* and *Blautia* as well as augmented *Eubacterium*, *Bifodobacterium* and *Christensenellacea*_R-7_group in PD patients receiving entacapone combined with L-dopa versus those treated with L-dopa alone [200]. 

Finally, GM can also modify the action of some drugs useful within the context of PD [201,202]. For instance, the propionate produced by the GM appears necessary to mediate the benefits of the osteoblast-secreted protein osteocalcin, which improves PD motor and non-motor symptoms [203]. Further, upon oral berberine intake, the enterococcal TH enzyme synthetizes L-dopa, which is then converted to dopamine in the brain, thus improving cognitive symptoms [204]. Overall, although the relationship between GM and medications is intriguing, new research is needed to better delve into this mutual influence and to exploit it for therapeutic purposes. 

## 4. Parkinson’s Disease and Gut Microbiota: Therapeutic Approaches

### 4.1. Gut Microbiota-Based PD Interventions: Antibiotics

Antibiotics are chemical compounds able to kill or arrest the growth of certain microorganisms. Although they are mainly used to counteract or prevent bacterial infections, their additional anti-inflammatory, immunomodulator, neuroprotective, antiamyloidogenic and antioxidant properties are becoming of increasing interest in the context of neurological disorders, including neurodegeneration [205,206,207,208,209]. Indeed, beside counteracting dysbiosis and constipation [210], it has been demonstrated that certain antibiotics can inhibit the activity of matrix metalloproteinases and prevent mitochondria dysfunction, microglia activation, protein misfolding and α-synuclein aggregation [211,212,213,214,215]. For example, treating mice where PD has been induced by MPTP with a cocktail of broad-spectrum antibiotics (ampicillin, metronidazole, and neomycin sulfate) was found to preserve TH and dopamine transporter immunoreactivities, which are generally lost upon MPTP administration [216]. This beneficial effect is mediated by an increase in *Proteobacteria*, as well as by a decrease in *Deferribacteres* and *Saccharibacteria* (*TM7)* abundance, which reflect an altered GM composition characterized by diversity loss [216]. Similar results were obtained in 6-OHDA-induced PD rats upon chronic treatment with an antibiotic mixture containing neomycin, pimaricin, bacitracin and vancomycin, which prevented dopaminergic neuronal death, relieved inflammation, ameliorated neurotoxicity and reduced motor impairments as measured by cylinder, rotation and stepping tests [217]. Recently, Cui et al. reported that vancomycin pretreatment of MPTP-induced PD mice improved motor symptoms by reducing SN astrocytes and microglia activation [218]. Notably, the authors proposed that neuroinflammation is indirectly inhibited by *Akkermansia* and *Blautia*, which increase in abundance upon vancomycin treatment and interfere with the toll like receptor 4 (TLR-4)/NF-κB pathway in the gut and in the brain [218]. Although *Akkermansia* is generally reported as harmful in PD patients, its dual negative and positive role may lean towards the latter when mucin conversion into SCFAs prevails over gut-barrier degradation, thus explaining this apparent discrepancy (see also Section 2.1 Gut microbiota dysregulation in PD). In humans, an intestinal decontamination therapy consisting of sodium phosphate enema, oral rifaximin and polyethylene glycol resulted effective in reducing dyskinesia and motor fluctuations related to PD, but more studies are required [219]. Other approaches focused on the use of certain specific antibiotics instead of cocktails have also been proposed to maximize the therapeutic benefit without impacting beneficial bacteria.

Rifaximin is a broad spectrum antibiotic with poor systemic absorption indicated to treat SIBO [210,220,221]. In this respect, rifaximin-mediated SIBO eradication in PD patients resulted in reduced motor fluctuations without impacting on L-dopa treatment [222]. This benefit should be ascribed to rifaximin-mediated modulation of the brain thyrotropin releasing hormone (THR) and THR-like peptides, which have caloric-restriction-like, anti-aging, neuroprotective properties and are known to be involved in the gut-brain axis [223]. However, no improvement in GI symptoms in 8 PD patients treated with rifaximin poses controversy over the actual efficacy of this antibiotic as PD treatment, calling for new studies [224].

Ceftriaxone (CTX) is a β-lactam antibiotic with a strong and safe past record [225,226]. The treatment of several PD animal models with CTX is known to improve neuroinflammatory and oxidative stress markers, stimulate neurogenesis and promote astrocyte viability through the suppression of NF-κB/c-Jun-mediated signaling [225,226,227,228]. Mechanistically, CTX also reduces extracellular glutamate levels by increasing the expression of the glutamate transporter-1 in astrocytes, thus avoiding brain excitotoxicity [226,228,229]. Moreover, it has been observed that CTX binds to α-synuclein with considerable affinity and prevents its polymerization in vitro [226,230,231]. in vivo, there is evidence that CTX treatment modifies the GM composition of MPTP-induced PD mice by disadvantaging the growth of *Proteus* while increasing the relative abundance of *Akkermansia* species, which act as probiotics when their SCFAs-converting activity exceeds that of intestinal barrier degradation (see also Section 2.1 Gut microbiota dysregulation in PD) [232]. 

Further studies proved the ability of CTX to reduce the levels of the main pro-inflammatory mediators TLR-4, MyD88 (myeloid differentiation primary response 88), IL-1β, TNF-α and NF-κB in the brain, TLR-4, MyD88, and NF-κB in the colon and IL-1β, TNF-α and IL-6 in the serum [232,233,234]. Similarly, CTX-mediated increase in the main antioxidant modulators glutathione, superoxide dismutase (SOD) and catalase was found to prevent the oxidative damage observed in rats treated with MPTP [233,234]. In line with these data, CTX administration was associated with reduced glial fibrillary acid protein (GFAP) and ionized calcium-binding adapter molecule 1 (IBA-1) expression, two markers of astrogliosis and microglia activation, respectively [232,235,236,237]. At the neuronal level, pre- or post- treatment with CTX prevented the loss of TH-positive neurons, reduced glutamatergic hyperactivity, and promoted neurogenesis at the level of SN and hippocampal dentate gyrus in different rat models of the disease [233,237,238,239,240,241,242]. As a consequence, dyskinesia, motor impairment and memory loss were all reverted upon CTX administration [233,234,237,238,239,240,242,243,244], although conflicting evidence still remains about its ability to improve learning outcomes [245]. Of note, CTX has been shown to interact synergistically with other compounds currently used or under investigation for the treatment of PD, such as erythropoietin, ropinirole and memantine, but the safety as well as the efficacy of these combinations should be further assessed [233,234,246]. 

Minocycline is a second-generation semisynthetic tetracycline with anti-microbial, anti-apoptotic, anti-inflammatory and antioxidant properties [247,248,249,250]. Thanks to the ability to efficiently cross the BBB, minocycline is considered neuroprotective for a variety of neurological conditions, including PD [251,252,253,254,255,256,257]. This effect is mainly ascribable to the minocycline-dependent suppression of microglia activation, which has been reported by several in vivo studies [251,258,259,260,261,262,263]. In this respect, microglial inactivation by minocycline correlates with decreased IL-1β formation, as well as reduced NADPH-oxidase and inducible nitric oxide synthase (iNOS) activity, suggesting that both anti-inflammatory and antioxidant pathways are involved [262,264]. In vitro, minocycline addition to 6-OHDA treated PC12 cells suppresses the release of lactate dehydrogenase, reactive oxygen species (ROS) and caspase 3 while supporting the activity of the antioxidant enzymes SOD and catalase [251,265,266,267]. Of note, these molecular changes seem to explain the increased striatal dopamine levels as well as the cognitive and locomotor improvements observed in zebrafish, mouse, and rat models [259,260,264,268,269,270,271]. Another mechanism through which minocycline prevents apoptosis is by limiting mitochondria dysfunction, inhibiting caspase 1 and 3 expression, and preventing the degradation of the antiapoptotic protein ICAD (the inhibitor of the caspase-activated deoxyribonuclease) [251,272,273,274]. However, despite the promising results, controversy remains. Indeed, an enhanced toxicity has been reported upon minocycline administration to MPTP-treated rodents and primates, resulting in disease exacerbation [275,276]. Moreover, results from a phase II clinical trial show no benefit from the use of minocycline and evidence decreased tolerability, although more studies are needed before drawing premature conclusions [277,278]. 

Doxycycline (DOX) is another broad-spectrum antibiotic belonging to tetracyclines that has been considered as PD treatment [279]. In vitro, DOX has shown anti-inflammatory properties by interfering with p38 MAP kinase and NF-κB pathways, reducing the expression of the activated microglia marker IBA-1 and inhibiting the production of the pro-oxidant and pro-inflammatory factors ROS, nitric oxide, iNOS, cyclooxygenase-2 (COX-2), IL-1β and TNF-α [280,281,282]. Concerning neuroprotection, DOX exerts an anti-apoptotic activity by repressing the matrix metallopeptidase-3 (MMP-3) in dopaminergic neurons and microglia both in vitro and in vivo [281]. In addition, DOX stimulates neurite growth through the activation of PI3K/Akt and MAPK/ERK pathways, independently from nerve growth factor activity [283]. Of note, recent studies demonstrated that DOX reduces the size and load of α-synuclein oligomers by converting them into high-molecular weight species that are not able to form fibrils, thus increasing cell viability [282,284]. When tested in vivo, DOX confirmed its neuroprotective activity by limiting dopaminergic neuronal loss in SN while increasing striatal dopamine levels [285,286]. This beneficial function is achieved by contrasting glial reactivity and by reducing the major histocompatibility complex-II expression in microglial cells [285,286]. In 6-OHDA-treated rats, both DOX and its derivative COL-3 showed an anti-dyskinetic potential when administered in combination with L-dopa [287]. According to the authors, the reduced levels of MMP-2/-9, MMP-3, ROS and of the dyskinesia-linked immunoreactivity markers FOSB, COX-2, GFAP and OX-42 would explain these benefits [287]. Nevertheless, despite promising in vivo data, clinical evidence is still lacking. 

Rifampicin is a macrocyclic antibiotic with cytoprotective functions that have been considered for PD treatment [288,289]. Indeed, there is evidence that rifampicin prevents α-synuclein fibrillation by promoting SUMOylation, which increases fibril solubility preventing neuronal death [290,291,292]. Other studies reported a reduction in IL-1β, TNF-α, IL-6 and ROS released by cells double treated with rotenone and rifampicin, thus indicating a promotion of neuroprotection [293,294,295]. Although not completely defined, rifampicin appears to sustain cell viability through different mechanisms: (i) by enhancing autophagy [293,295]; (ii) via PI3K/Akt/GSK-3β/CREB pathway modulation [296]; (iii) by upregulating the unfolded protein response marker GRP78 through the PERK/eIF2α/ATF4 pathway [297]. In vivo, MPTP-induced PD mice treated with rifampicin showed increased striatal and SN TH immunoreactivity, attenuated levels of oxidative stress and re-established dopaminergic signaling in the striatum [298]. More recently, rotenone-induced PD in zebrafish has shown benefit from rifampicin administration due to the decrease in neuroinflammation [299].

Generally, although promising, two main concerns remain about the use of antibiotics in PD treatment: (i) antibiotics can kill some specific microbial populations leading to intestinal dysbiosis and neurological dysfunction and (ii) their prolonged and widespread intake would favor antibiotic resistance [213,300,301]. There is evidence that ceftriaxone (a third-generation cephalosporin)-induced dysbiosis worsens motor symptoms in 6-OHDA treated mice and correlates with dopaminergic neuron toxicity as well as intestinal and systemic inflammation [302]. Moreover, quinolones and β-lactams are known to trigger neurotoxicity through their interference with gamma-aminobutyric acid and benzodiazepine receptors signaling [213]. Mechanistically, it has been hypothesized that antibiotic-induced dysbiosis may favor the growth of *Enterobacteria* producing the bacterial α-synuclein curli, which promotes neurodegeneration [303,304]. In addition, leaky gut-mediated systemic inflammation might result from dysbiosis and mediate the BBB damage, allowing circulating neurotoxins to enter the brain [305]. In humans, a Finnish study conducted on 13,976 PD and 40,697 healthy individuals showed that taking certain antibiotics years earlier, especially macrolides and lincosamides, correlates with an increased risk of developing PD [306]. However, results from another prospective study involving 59,637 women did not report any correlation between antibiotic intake and PD incidence [307]. Overall, contrasting results and scarce long-term safety data remain a concern. Innovative drug delivery systems based on nanoparticles are now being tested to improve the clinical benefit of these antibiotics [279]. At the same time, synthetic tailoring to potentiate the neuroprotective chemical functions over the antimicrobial ones is another promising approach for the risk-benefit optimization [301].

### 4.2. Gut Microbiota-Based PD Interventions: Probiotics

Probiotics are defined as “live microorganisms that, when administered in adequate amounts, confer a health benefit on the host” [308]. It is widely reported that the most common bacteria used as probiotics (Lactobacilli, Bifidobacteria, and Enterococci) [309] have potential benefits in restoring the GM, reducing intestinal permeability, inflammation, and oxidative stress, improving immune homeostasis and GI symptoms (constipation, diarrhoea, bloating, and abdominal pain), as well as preventing or counteracting several conditions, including GI, liver, and cardiovascular diseases, obesity, diabetes, cancer, and H. pylori and urogenital infections [215,310,311,312,313]. Moreover, it is now evident that GM dysbiosis is a factor that takes part in the development of several neurological diseases, including PD, AD, multiple sclerosis, autism spectrum disorders (ASD), anxiety, depression, schizophrenia, and other mental illnesses [314,315]. Concerning PD, as previously mentioned, altered GM could contribute to the onset of some PD-related complications, such as constipation, the most common non-motor symptom [316]. Therefore, modulation of the microbiota-gut-brain axis using probiotics could be a promising complementary approach to traditional methods to prevent or counteract these disorders, including PD, as widely reported in literature [315,317,318,319,320]. For instance, Bacteroides fragilis has been documented to improve ASD symptoms and gut barrier integrity, and reduce intestinal permeability [321]; further, the probiotic SLAB51, a formulation of nine live bacterial strains (Streptococcus thermophilus, *B. longum*, *B. breve*, *B. infantis*, *L. acidophilus*, *L. plantarum*, *L. paracasei*, *L. delbrueckii* subsp. *bulgaricus*, and *L. brevis*) improves cognition and reduces the accumulation of amyloid plaques, brain injury, and inflammatory cytokines plasma levels in AD mice [322], while the assumption of a probiotic fermented milk drink containing *L. acidophilus*, *L. casei*, *B. bifidum*, and *L. fermentum* improves cognitive function in AD patients [323]. Concerning PD, many studies showed that probiotic intake can reduce neuroinflammation, inhibit the loss of dopaminergic neurons, and modulate brain functions, as explained in the sections that follow [324,325,326,327].

#### 4.2.1. Preclinical Studies on Probiotics Supplementation in PD

Limited in vitro experiments have been carried out to evaluate the possible beneficial and neuroprotective effects of probiotics in alleviating the typical features of PD (Table 1) [325,328,329,330,331,332,333,334,335,336,337,338,339,340,341,342]. 

An in vitro GM model created with stool samples from PD patients and a cell culture model (Caco-2/THP1 cells) has been used to study the benefits of Symprove™ (*Lactobacillus acidophilus* NCIMB 30175, *L. plantarum* NCIMB 30173, *L. rhamnosus* NCIMB 30174 and *Enterococcus faecium* NCIMB 30176). Ghyselinck et al. found that the treatment with this multi-strain probiotic can change the bacterial composition (increase in *Firmicutes* and decrease in *Bacteroidetes*), stimulate the production of SCFAs and lactate, modulate mucosal inflammation (by increasing anti-inflammatory cytokines such as IL-6, IL-10 and decreasing the pro-inflammatory chemokine IL-8), and improve intestinal permeability [329]. Another study, performed by using peripheral blood mononuclear cells isolated from PD patients, Caco-2 cells, and *Escherichia coli* and *Klebsiella pneumoniae* inoculation, showed the potential use of *L. salivarius* LS01 and *L. acidophilus* LA02 in modulating inflammation (reduction in TNF-α, IL-6, and IL-17A, and increase in IL-4 and IL-10), oxidative stress, and gut permeability, and inhibiting the proliferation of pathogenic bacteria (*E. coli* and *K. pneumoniae*) [328]. In addition, Cheon et al. reported the neuroprotective effects of the heat-killed *L. plantarum* 200655 on H_2_O_2_-treated SH-SY5Y human neuroblastoma cells. Indeed, this probiotic is able to increase the brain-derived neurotrophic factor (BDNF) and TH mRNA expression, and decrease apoptosis [330]. Further, Castelli et al. investigated the effects of the probiotic formulation SLAB51 on the SH-SY5Y cell model of PD finding a reduction in dopaminergic neuronal loss in the SN and striatum; this effect was associated with a rise in the activation of the neuroprotective and neuronal survival BDNF pathway, a reduction in the neuronal death pathway, and a significant decrease in 4-hydroxynonenal protein adducts level, suggesting its potential antioxidant property [331].

Interestingly, Surwase and Jadhav observed the ability of the probiotic *Bacillus* sp. JPJ to synthesize L-dopa from L-tyrosine in vitro [343]; moreover, some probiotic strains (especially *Enterococcus* and *Lactobacillus*) have dopa decarboxylase genes in their genome, hence they can convert L-dopa to dopamine through this bacterial enzyme [180]. This evidence suggests that the combination of probiotics and L-dopa could represent a more efficient therapy for PD, although it is unlikely that dopamine may reach, as such, the brain due to the BBB presence [29].

Overall, these in vitro studies highlight the helpful role of specific probiotics in PD models; however, in vivo studies, with the same probiotics, are mandatory to support these positive results and help to better understand their safety and efficacy. Nevertheless, within this context, numerous studies, performed also by using animal models of PD, report the potential beneficial effects of probiotics supplementation on clinical symptoms and biochemical markers (Table 1). For instance, in a murine model of PD, Wang et al. demonstrated the neuroprotective role of *L. plantarum* DP189. Indeed, they reported the ability of this probiotic to reduce the aggregates of α-synuclein in the SN through the modulation of oxidative stress, inflammation, and GM dysbiosis. In this regard, they observed a rise in the abundance of *Lactobacillus* and *Prevotella* and a reduction in the content of *Proteobacteria* and *Actinobacteria* [335]. Furthermore, in a *Caenorhabditis elegans* model, it has been shown that also the probiotic *Bacillus subtilis* PXN21 can inhibit α-synuclein aggregation and promote the clearance of preformed aggregates [344]. In addition, a mixture of probiotics (*L. plantarum* CRL 2130, *S. thermophilus* CRL 807, and *S. thermophilus* CRL 808) has been shown to improve motor behaviour and neuroinflammation in a murine model of PD [345], while *L. salivarius* AP-32 enhanced the activity of antioxidant enzymes [SOD, glutathione peroxidase (GPx), and catalase] in a rat model of PD [346]. Another study, carried out in the 6-OHDA PD rat model, reports that supplementation with a mixture of probiotics (*L. acidophilus*, *B. bifidum*, *L. reuteri*, and *L. fermentum*) improves rotational behaviour and memory dysfunction, reduces the number of injured neurons and lipid peroxidation by decreasing malondialdehyde levels [347]. Moreover, treatment with *Lacticaseibacillus rhamnosus* HA-114 can improve cognition deficits but has no effects on anxiety-like behaviour in the 6-OHDA PD rat model [348].

Interestingly, creating probiotics genetically manipulated could be a strategy to increase their beneficial effects. For instance, by using a murine model of PD, it has been shown that the oral administration of the engineered strain *Lactococcus lactis cremori* that continually expresses glucagon-like peptide-1 (GLP-1) (MG1363-pMG36e-GLP-1) can increase TH expression, reduce locomotor and memory impairments, as well as α-synuclein production, attenuate neuroinflammation via down-regulating the TLR4/NF-κB pathway and some pro-inflammatory cytokines (IL-1β, IL-6, and TNF-α), arrest microglia and astrocyte activation, and restore GM dysbiosis [349,350]. Of note, GLP-1, crossing the BBB, binds to GLP-1 receptors, thus activating in the brain the insulin-signaling pathway involved in neurogenesis, synaptic plasticity, and neuronal metabolism [351]. 

Nevertheless, despite preclinical evidence suggests the benefits of probiotic supplementation in PD, in a murine model of PD, Dwyer et al. found that VSL#3 (a mixture of eight live bacterial strains) had no significant effects in modifying GM composition, preventing the reduction of dopaminergic neurons, and modulating inflammation [352,353]. These conflicting results emphasize the need of more studies aimed to better ascertain which specific probiotic supplementation, most likely a multi-strain mixture, can provide a more successful therapeutic support to face PD. 

#### 4.2.2. Clinical Studies on Probiotics Supplementation in PD

Human clinical trials have demonstrated the possible use of probiotic supplements as a potential therapeutic adjuvant for the treatment of PD. They could represent an alternative and complementary method to the traditional treatment approaches, useful to manage the disease and alleviate some of the common symptoms (Table 1). 

A pilot study, performed by administering *L. plantarum* PS128 together with L-dopa, reports the ability of this probiotic to improve UPDRS motor scores and quality of life of PD patients, and to reduce plasma myeloperoxidase and urine creatinine levels, despite no significant changes found in non-motor symptoms [354]. Conversely, some double-blind, randomized, placebo-controlled single centre trials reported the efficacy of multi-strain probiotics in improving non-motor symptoms, including GI motility, stool consistency, and quality of life in PD patients with constipation [340,341]. In addition, Cassani et al. showed that consumption of fermented milk with *L. casei* Shirota can reduce abdominal pain and bloating, and ameliorate stool consistency and spontaneous defecation [336], while Georgescu et al., administering *L. acidophilus* and *B. infantis* to older PD patients, observed an improvement in abdominal pain and bloating [337]. In another randomized, double-blind, placebo-controlled clinical trial PD patients were treated with *L. acidophilus*, *L. reuteri*, *L. fermentum*, and *B. bifidum*. The obtained results show a reduction in UPDRS motor scores [339]. Conversely, the study conducted by Borzabadi et al. reported unaltered UPDRS motor scores and Non-Motor Symptom Scale (NMSS) scores in patients treated with probiotics versus the placebo cohort. Nevertheless, they observed, compared to placebo, a significant reduction in cytokines involved in inflammation (IL-1, IL-8 and TNF-α) [338]. Finally, another randomized, double-blind, placebo-controlled clinical trial, performed by using the probiotic strain Probio-M8 (*B. animalis* subsp. *lactis* Probio M-8), together with conventional drugs (benserazide and dopamine agonists), showed amelioration in sleep quality, cognitive dysfunction, defecation, and attenuation of GI symptoms [342].

In addition to being an effective support to manage some pathological conditions, probiotics can also be used as prevention tools. As an example, some evidence suggests that, during adolescence, environmental factors, such as stress, infections, inflammation, and use of antibiotics, can lead to dysbiosis, resulting in the risk to develop neurological disorders related to brain aging in adulthood. Therefore, the consumption of probiotics already from adolescence could be a good approach to prevent GM alteration and protect everyone against the onset of neurodegenerative diseases, such as PD [355].

Interestingly, some innovative approaches are also emerging from the technological/formulative point of view that could be exploited to improve the efficacy of the treatment [356,357]. For instance, Enck et al. designed a novel system for the delivery of probiotics. They encapsulated bacterial cells in modified alginate hydrogel, in order to protect the bacteria from the degradation by the acidic gastric environment [356]. 

In conclusion, despite the emerging benefits, the studies are still limited. Future extensive and long-term experiments are necessary to: (i) confirm the positive results obtained until now, (ii) investigate the precise mechanisms of action underlying probiotics effects, and (iii) obtain more knowledge on GM composition in different populations of patients, to find the best bacterial strains to be used, and define dosage, duration of administration, and the possible combination of different approaches. 

### 4.3. Gut Microbiota-Based PD Interventions: Prebiotics

According to the International Scientific Association for Probiotics and Prebiotics (ISAPP), prebiotics are defined as “substrates selectively used by host microorganisms that confer health benefits to the host, while retaining the microflora-mediated health benefits” [358]. Prebiotics are dietary fibres originated from soybeans, raw oats, unrefined wheat and barley, non-digestible carbohydrates and oligosaccharides, including galacto-oligosaccharides (GOS), fructo-oligosaccharides (FOS), inulin, and lactulose [359,360]. Polyphenols (catechin, epicatechin and quercetin) can also act as prebiotics [361]. They can alter GM composition, by favouring the growth and the activity of beneficial bacteria, and by decreasing pathogens in the GI tract; further, they have positive effects on lipid metabolism, decrease the recurrence of Clostridium difficile infections, and alleviate GI and allergic disorders [362,363,364].

In the gut, the beneficial microbes metabolize the prebiotics, resulting in the generation of SCFAs (namely, acetate, propionate, butyrate) that are involved in neuromodulation, in anti-inflammatory processes, in the regulation of both intestinal and blood-brain barriers [365,366]. 

Like probiotics, prebiotics also play a beneficial role in managing neurological and neurodegenerative diseases [367]. For instance, lactulose and melibiose improve short-term memory and cognitive ability in AD mice [368]; bimuno-GOS ameliorate anti-social behavior in children with ASD [369]; oral administration of *Marinda officinalis*-derived oligosaccharides ameliorates memory and learning ability, decreases plaque formation, oxidative stress, and inflammation in both rats and mice AD models [370,371].

Concerning PD, to date, few studies have been conducted to evaluate the effects of prebiotics on PD animal models and patients (Table 2) [316,346,372,373,374,375]. In a mouse model of PD, Perez–Pardo et al. found that prebiotic fibers (FOS, GOS and nutriose, a soluble corn fibre) can normalize motor symptoms, reduce α-synuclein levels, and restore GI dysfunction, inflammation and dopamine transporter expression [372]; further, it has been shown that the prebiotic polymannuronic acid can prevent dopaminergic neuronal loss via SCFAs-mediated anti-inflammatory and anti-apoptotic mechanisms [373]. In addition, another study, performed by using 6-OHDA PD rat model, reported that the supplementation with the medium obtained from the probiotic *L. salivarius* subsp. *salicinium* AP-32 culture can reduce dopaminergic neuronal loss, motor dysfunctions, muscle atrophy, oxidative stress (increased SOD and GPx) and inflammation [346,376]. Interestingly, another study highlighted a raise in BDNF levels in the hippocampus of rats after the administration of FOS and GOS [377]. Since BDNF is involved in neuronal protection, survival, growth, and in synaptic plasticity [378], this finding suggests that prebiotics supplementation might have a role on brain neuroprotection. Finally, some studies performed in PD animal models report the beneficial effects of sodium butyrate in improving PD symptoms [379,380]; therefore, butyrogenic prebiotics could be used to increase butyrate concentration in the colon and help to manage PD [365].

In patients, two studies reported the effects of insoluble fibers on constipation. Indeed, both Astarloa et al., by administering wheat, pectin, and dimethylpolyoxyhexane-900, and Ashraf et al., by using psyllium, found a significant improvement in constipation [374,375]. Finally, another study investigated the effects of an oral supplementation with resistant starch, whose fermentation by anaerobic bacteria leads to the production of SCFAs, finding an increased butyrate concentration, as well as an improvement in non-motor symptoms [316]. 

In conclusion, despite few studies on PD, the satisfactory clinical outcomes on patients, especially on constipation, suggest that prebiotics might be a possible adjuvant therapy for PD, although more human clinical trials are mandatory to support this conclusion.

### 4.4. Gut Microbiota-Based PD Interventions: Diet

Although multifactorial interactions are involved in the prevalence and incidence of neurodegenerative diseases, nutrition plays an essential role in the pathogenesis and development of neurodegenerative diseases such as AD and PD [381,382]. Recent findings have revealed that diet, as a non-pharmacological element, plays an important role not only as a risk factor but also as a potential therapeutic approach for treating PD (Table 3) [309,383,384,385,386,387,388,389,390,391,392,393,394,395,396,397,398]. The effects of diet intervention on PD development can be attributed to different mechanisms. First, by altering intestinal microbiota composition and consequently affecting the gut-brain axis or by directly interfering with immune cells. As a matter of fact, diet is probably the most influential factor in determining the structure and metabolic function of the intestinal microbiota. Moreover, dietary components might also modulate the chronic inflammatory response that is associated with aging. Intriguingly, diet components can reduce constipation and improve L-dopa uptake, which is the first-line therapy for PD [399,400]. Therefore, consuming a constant diet on a long-term basis can impact the development of PD; however, it is still to be elucidated as to how a particular diet reduces the risk of this development. Here, we discuss how changes in diet may prevent or modify PD progression, with a special focus on Mediterranean, ketogenic, and omega-3-rich diets. 

#### 4.4.1. Mediterranean Diet

There has been extensive research on the Mediterranean diet (MD) over the years, and one large systematic review indicated that it is associated with a reduced incidence of cancer, cardiovascular disease, and AD [401]. High consumption of plant foods, including vegetables, nuts, fruits, and whole grain, moderate to weekly consumption of fish, poultry, eggs, and red wine, high intake of unsaturated fatty acids (mainly in the form of olive oil), a low to moderate intake of dairy products, and a limited use of saturated fatty acid, as well as red meat, are the major cornerstones of MD [384,387,400]. Of interest, adherence to MD is associated with a decreased risk of PD development [389,390,391,402]. One major component of MD is the high intake of dietary fibers (around 30 g/day) [403], which in turn can be utilized by the GM (especially by SCFAs-producing bacteria) as a source of energy [404]. Dietary fibers alter the gut microbiota composition in favor of fiber-fermenting bacteria rather than Gram-negative (LPS-producing) bacteria [405,406], leading to decreased neuroinflammation in PD [166,407]. Consuming a high-fiber diet may therefore improve intestinal barrier function, insulin resistance and sensitivity, GLP-1 and BDNF levels, all of which may contribute to slowing the progression of PD [166,408,409,410].

In addition to fiber, MD also contains a high quantity of flavonoid antioxidants, especially polyphenols, which have been associated with a reduced risk of PD [384,388,411]. Flavonoids in MD are present in fruits, vegetables, grains, olives, and tea. Each flavonoid molecule may act simultaneously on different mechanisms, which promote the restoration of oxidative homeostasis, the reduction of the neuroinflammatory process, and the enhancement of α-synuclein aggregates clearance [412,413,414]. Moreover, the potential of polyphenols as antioxidants and anti-inflammatory agents, as well as their ability to improve endothelial function, may contribute to lowering the risk of developing PD [411,415,416,417,418].

On the other hand, one of the features of MD is the moderate intake of dairy products. In this regard, a comprehensive umbrella study shows that high consumption of total dairy foods compared to a low intake is associated with an increased risk of PD [419]. A possible explanation is that milk proteins (casein and lactalbumin) lower serum urate levels, where urate may play a protective role against PD [420,421]. It has also been suggested that the pesticide content in dairy foods may play a role: specifically, it has been suggested that genetic susceptibility to pesticide metabolism, elimination, and transport, as well as mitochondrial dysfunction, oxidative stress, and neuronal loss, could all promote PD [422]. 

Another type of diet, known as the Dietary Approaches to Stop Hypertension (DASH), aims to treat and prevent high blood pressure. DASH diet shares many of the same principles as MD. Recently, some experts have suggested combining Mediterranean and DASH diets to boost cognitive function. This approach, named Mediterranean-DASH Intervention for Neurodegenerative Delay or MIND diet, has the potential to postpone the decline of cognitive scores in patients with neurodegenerative diseases [423]. Interestingly, the findings of a cross-sectional study revealed that MIND diet adherence is associated with an older age of PD onset in a superior manner to that of the MD itself [383]. Furthermore, this diet may improve fatigue and depression in PD patients [393].

In conclusion, although still limited, these observations support the need for conducting randomized controlled trials in PD patients to determine whether MD or MIND can influence neuroinflammation or the course of PD and, if so, which components provide the most benefit.

#### 4.4.2. Ketogenic Diet

The ketogenic diet (KD) is defined as a high-fat (70–80% fat from total daily calories), adequate-protein (10–20%), low-carbohydrate (5–10%) intake [424]. As a result of such changes in macronutrient proportions, a process called ketosis results, which allows glucose to be replaced by ketone bodies in the form of acetoacetic acid, β-hydroxybutyrate (BHB), and acetone [425,426]. There have been several studies on animal models of PD showing the benefits of ketone bodies [427,428,429,430], but only a limited number of human studies have been conducted [395,396]. In this regard, the UPDRS scores greatly improved for five patients following a KD for 4 weeks [395]. In addition, a 3-month KD was also shown to improve the voice handicap index in PD patients in comparison to a regular diet [394]. Furthermore, Phillips and colleagues [396] conducted an 8-week experiment comparing a low-fat, high-carbohydrate diet with a KD. Although motor and non-motor symptoms improved in both diet groups, non-motor symptoms, such as cognitive function, ameliorated more in the KD group.

Likewise, in a rodent model of PD, BHB protects dopaminergic neurons from damage triggered by MPTP [431]. Moreover, BHB injections into the brain of mice can ameliorate the symptoms of MPTP-induced dopaminergic neurodegeneration and motor deficits [430]. Ketone bodies can exert their beneficial effects through a variety of mechanisms. For instance, adenosine triphosphate (ATP) production by ketones increases mitochondrial respiration, thus providing a neuroprotective effect [432]. It should be also mentioned that ketone bodies such as BHB provide the brain with more energy per unit of oxygen than glucose. In any case, these processes give rise to enhanced potassium channels activity, which is sensitive to ATP and adenosine, increased neurotrophic factor expression, expanded energy reserves, and stabilization of neuronal action potentials that lead to an improvement in PD symptoms [425,433]. Another intriguing potential mechanism is the effect of ketone bodies on CNS inflammation. These compounds can cross the BBB, inhibit the inflammatory response by up-regulating anti-inflammatory genes, such as MAP3K8 and TLR5, and by down-regulating pro-inflammatory genes such as TNFSF6, TNF-α, and nuclear factor-κB (NF-κB), thus decreasing inflammatory factors such as interleukins (IL-1β, IL-6) and TNF-α in SN and reducing microglia activation in various animal and in vitro models [434,435,436,437,438].

On the flip side, any diet intervention may potentially also affect PD’s pharmacotherapy. To the best of our knowledge, just one study investigated the effects of KD on L-dopa (as mentioned, one of the gold standard treatments for PD) properties. However, the findings of this study indicate that KD does not significantly influence the pharmacokinetics and pharmacodynamics of L-dopa [439].

Once again, although it is a must to investigate the detailed mechanism of action of KD’s components, as well as the overall effectiveness of this diet in large randomized clinical trials and cohort studies, it must be highlighted that patient compliance with the KD (even when modified) is poor, due to the restrictive nature of the diet and GI discomfort symptoms.

#### 4.4.3. Omega-3 Fatty Acids

Omega-3 (ω3) polyunsaturated fatty acids (PUFAs), including docosahexaenoic acid [DHA, 22:6 (n-3)], eicosapentaenoic acid [EPA, 20:5(n-3)], and α-linolenic acid [ALA, 18:3 (n-3)], play a crucial role in maintaining the structure and healthy function of different organs. They are involved in inflammatory and immunological processes, as well as in hormonal regulation [440,441]. Some limited human studies have shown that consuming PUFAs, being components of different diets such as MD or KD, and specifically the supplementation with EPA and DHA may reduce the risk of PD and relieve some of its symptoms like motor symptoms [442,443,444]. Besides the motor symptoms, depression affects over 40% of PD cases [445]. According to both epidemiological and clinical studies, a reduced dietary intake of fatty acids, especially ω3, is associated with mood disorders and depression [446,447,448,449]. Interestingly, in a clinical study where PD patients were taking fish oil (a great source of ω3) with or without antidepressants presented improvements in depressive symptoms [450]. As a result, the addition of ω3 to the diet of PD patients may be a valuable approach to reduce depression symptoms, which can also impact other clinical aspects of the disease.

Besides having a crucial role in membrane fluidity and a broad spectrum of activities, ω3 may be also beneficial at several levels of the neuronal degenerative process observed in PD [451]. As mentioned, PD, like other neurodegenerative diseases, is associated with oxidative stress and inflammation pathways, which are tightly linked and interdependent [452]. Elevated levels of cytokines and prostaglandins have been detected in the cerebrospinal fluid and brains of PD patients where they may in turn activate the microglia, induce migration of microglia to SN, and release pro-inflammatory cytokines, as well as neurotoxic molecules capable of further exacerbating the disease [453,454,455,456]. Within this context, ω3 are able to ameliorate oxidative stress and neuroinflammation, which may explain part of their beneficial effects on PD [457]. Indeed, a clinical study found that supplementation of PD patients with ω3 has a favorable effect on glutathione concentrations, as well as on the total antioxidant capacity, and it is associated with a lower amount of C-reactive protein [458]. These findings are supported by experimental evidence that ω3-PUFAs reduce NOS activity and increase BDNF levels in the CNS [459,460]. In addition to their neuroprotective effects, ω3 (especially DHA) may modulate the brain’s dopamine systems through different mechanisms [461]. In conclusion, these observations provide strong scientific support for conducting randomized controlled trials to assess whether ω3 supplements can slow down degeneration and thereby modify the course of the disease.

### 4.5. Gut Microbiota-Based PD Interventions: Fecal Microbiota Transplantation

Fecal microbiota transplantation (FMT) consists in the transfer of resuspended and filtered stool material from a healthy donor to a patient’s gut. The aim of this approach is to counteract dysbiosis while favoring the establishment of a beneficial and balanced microbiota [462,463]. Although colonoscopy is the preferred method of transplantation, delivery through nasogastric or nasojejunal tube, enema, or orally administered capsules have also been tested [462,464]. Following the successful use of FMT in the treatment of refractory or recurrent *Clostridium difficile* infection, several studies have been conducted to explore FMT as a therapeutic strategy for a wide range of neurological disorders, including multiple sclerosis, epilepsy, ASD, Tourette syndrome, diabetic neuropathy, AD and PD, with promising preclinical and clinical data [30,465,466,467,468]. Concerning PD, consistent preclinical studies and a handful of human case reports have shown that FMT might be exploited to reduce motor and non-motor symptoms, as well as constipation, at least in the short term [40,465,469,470,471,472,473,474,475,476,477] (Table 4). Early evidence came in 2016 from the work of Sampson et al., who first reported that the transfer of fecal matter from human PD patients to α-synuclein overexpressing mice substantially worsened their physical symptoms in comparison with mice receiving feces from healthy human donors [40]. These results were then confirmed in 2018, when Sun et al. showed that fecal microbiota transfer from PD mice to their healthy counterpart increases motor deficits while reducing the striatal levels of the neurotransmitters dopamine, serotonin and their metabolites, thus reproducing the typical features of the disease [469]. Conversely, fecal matter transplantation from healthy mice to PD recipient mice improved physical performance, ameliorated motor symptoms and reduced dysbiosis in several independent studies [469,470,471,472]. Looking at the GM composition, there is evidence that FMT re-establishes eubiosis by disadvantaging the growth of *Desulfovibrio*, *Akkermansia* and *Proteobacteria* (orders *Enterobacteriales* and *Turicibacteriales*), while simultaneously favoring the proliferation of beneficial bacteria such as *Bacteroidetes* and *Actinobacteria* phyla, with a particular effect on *Blautia* and *Prevotella* species [469,470,472]. Moreover, FMT appears to protect from gut inflammation by promoting intestinal barrier integrity and reducing the levels of LPS in the colon, serum, and SN, therefore preventing leaky gut and systemic inflammation [470]. At the brain level, FMT contrasts cognitive damage by decreasing α-synuclein expression and restoring the optimal levels of the striatal neurotransmitters dopamine and serotonin, thus supporting neuroprotection [469,471,473]. Notably, decreased neuroinflammation following FMT has been reported by numerous preclinical studies [469,470,471,472]. This beneficial effect should be ascribed to the ability of GM to modulate microglia and astrocyte activation in SN by regulating the TLR4/NF-κB pro-inflammatory pathway and reducing the expression of GSK3β, iNOS and IL-1β, which are implicated in PD pathogenesis and progression [469,470,471,472,478,479,480]. 

Nevertheless, FMT studies involving humans are still limited. One case study conducted in 2019 by Huang et al. investigated the potential therapeutic benefit of FMT in a 71-year-old male PD patient reporting constipation (>3 years) and motor symptoms (for 7 years). While FMT successfully promoted regular defecation, tremor disappeared only temporarily and then reappeared two months after transplant [474]. Three subsequent studies involving 6, 11 and 15 PD patients, respectively, confirmed the reduction in constipation, as well as in motor and non-motor symptoms following FMT, as indicated by the decreased scores registered in various PD assessment tests [475,476,477]. 

On the whole, from a clinical point of view, better and longer-term outcomes were obtained using colonoscopy compared to nasointestinal delivery [476]. In line with preclinical data, PD patients undergoing FMT showed an increased presence of *Blautia* and *Prevotella* and a diminished overall abundance of *Bacteroidetes*, thus confirming the efficacy of this approach in modifying the GM composition [475]. 

Despite the therapeutic potential of FMT for the treatment of PD, several limitations still exist and need to be addressed. Standard clinical protocols, delivery methods, periodicity, donor’s selection criteria, patient’s inclusion criteria, long-term benefits and potential risks remain an issue [463,467,481,482,483,484,485]. Within this context, 6 cases of adverse events occurred in human studies: flatulence (1), diarrhea (2), hospitalization under observation (1) and GI pain (2) [476,477]. Therefore, although not life-threatening, the nature of these complications should be better investigated. 

In addition, randomized controlled trials involving a considerable number of patients are required to better assess feasibility, therapeutic efficacy, safety and long-term benefits of this promising GM-modifying approach [486].

## 5. Oral and Nasal Microbiota: Other Important Districts Involved in the Disease

Preliminary evidence is showing that specific oral microbiota compositions, also known as oral microbiota signatures, are associated with human healthy aging [487]. Accordingly, alterations in the abundance of oral bacteria have been directly or indirectly linked to the onset of different disorders, such as cancer, cardiovascular and pulmonary diseases, stroke, diabetes and even neurodegeneration [487,488,489,490,491]. In the latter case, it has been hypothesized that the increased prevalence of mouth anaerobic microbes observed during aging might generate a TNF-α-mediated pro-inflammatory environment that damages the BBB integrity and favors bacteria spreading [492]. In this respect, 16S rRNA sequencing of the oral microbes performed in mild cognitive impaired (MCI) or healthy individuals revealed that increased *Pasteurellaceae* and decreased *Lautropia mirabilis* were associated with altered cerebrospinal fluid levels of the inflammatory mediators oncostatin M, T-cell surface glycoprotein CD8 alpha chain, MMP10, thymic stromal lymphopoietin and chemokine ligand 3 [493]. In AD, there is evidence that oral microbiota dysbiosis induced by alcohol consumption takes part in AD pathogenesis through the modulation of eIF2, eIF4 and mTOR pathways [494]. Moreover, results from a study conducted in a cohort of Canadian individuals with neurodegeneration reported higher microbial diversity (in contrast to what is observed in the GM), diminished *Streptococcaceae* and *Actinomycetaceae*, as well as enhanced *Weeksellaceae* and *Porphyromonas* in these patients compared to controls, but these changes did not correlate with cognitive decline [495]. Concerning PD, oral features associated with the disease such as dysphagia, salivary pH and drooling may influence the oral microbiota by altering the β-diversity index and favoring the growth of opportunistic oral microbes and *Lactobacillus* species, which may exacerbate the clinical symptoms and correlate with worse Hamilton Anxiety Scale and Hamilton Depression Rating Scale scores [70,496,497]. Moreover, combined oral-gut microbiome metagenomic sequencing revealed a link between oral *Lactobacillus* and gut opportunistic pathogens, suggesting a connection between these two microbial communities [498]. Other oral microbial species whose abundance has been found altered in PD patients are: *Actinomyces AFQC_s*, *Scardovia*, *Kingella oralis*, *Streptococcus mutans*, *Veillonella AFUJ_s*, *Prevotella*, *Firmicutes*, *Negativicutes* (all increased) and *Lachnospiraceae AM420052_s*, *Treponema KE332528_s*, *Proteobacteria*, *Tenercutes* and *Pastescibacteria* (all decreased) [499,500]. Of note, these alterations can modulate the expression of salivary host mRNAs involved in multiple brain activities, thus supporting the relationship between oral microbiota and cognitive disorders [497]. 

Because of dysbiosis, the aminoacidic and energetic metabolisms associated with oral bacteria vary [497]. In this respect, 16SrRNA sequencing performed on 91 PD patients and 91 control individuals reported an increase in pathways related to oxidative phosphorylation and carbohydrate metabolism among oral microbes associated with the disease [70]. Another consequence of dysbiosis is the establishment of a pro-inflammatory environment, in a closely interconnected feedback loop [70,501]. For example, an increase in different local pro-inflammatory molecules, such as IL1-RA, IL-1β and TNF-α, has been associated with dysbiosis in a study comparing 20 PD patients and 20 matched controls, which may contribute to the onset of PD [499]. Moreover, mice receiving *Porphyromonas gingivalis*, a pathogen associated with chronic periodontitis, showed sustained colon TNF-α and IL-1β expression, as well as higher levels of serum IL-17A and its receptor (IL-17AR) in the brain, which were associated with loss of dopaminergic neurons, α -synuclein accumulation and microglia activation [502]. However, more studies are needed to better address the role of oral dysbiosis in triggering local, intestinal and systemic inflammation, and to evaluate the impact of this highly interconnected pathway on brain function. 

Since olfactory dysfunction is an early marker of PD, which arises far before the appearance of the motor symptoms, few authors started to investigate the potential role of nasal microbiota in disease pathogenesis [503]. Given the interconnection between nose and brain, some studies hypothesized that changes in nasal microbiota community may promote neuroinflammation through the olfactory bulb, thus establishing a nose-to-brain axis [503,504]. However, until today, data remain scarce. So far, the only evidence comes from a study by Pal et al., who performed 16SrRNA sequencing of the deep nasal cavity in PD patients versus healthy controls and revealed a positive correlation between the abundance of the opportunistic bacterium *Moraxella catarrhalis* and motor symptoms in PD [503]. Nevertheless, no significant differences in nasal microbiota composition have been reported in two other studies comparing 91 PD patients to 91 controls and 76 PD patients to 78 healthy controls, respectively [70,80]. This discrepancy probably reflects the struggle to consider different variables, such as sampling location, collection method and analysis pipeline, which often differ among studies [503]. Moreover, the nasal microbiota composition, as well as the oral one, changes according to dietary habits, host factors, environmental conditions and even the season [503,505]. 

Overall, promising evidence is emerging for an association between oral/nasal microbiota and PD, but data remain limited and sometimes conflicting. Once better investigated, these two microbial communities might be exploited as early diagnostic and therapeutic tools for PD, as already proposed [504]. For example, the combination of 11 taxonomic features associated with the oral microbiota has allowed PD diagnosis reaching up to 84.5% accuracy, but better performances are expected to come [497]. Moreover, yeast and phage populations seem also to differ in PD patients compared to controls, and their further investigation might be of interest [497]. Until now, however, since the number of studies investigating the role of nasal and oral microbial communities on PD remains scarce, more data are needed before drawing any conclusions [506]. 

## 6. Discussion

Overall, our narrative review summarizes the main data on the link between dysbiosis and PD, with a particular focus on the possibility of taking advantage of this knowledge not only for diagnostic purposes, but also for a therapeutic application. However, several limitations still exist and need to be discussed. A major concern is the discrepancy and variability sometimes found in GM studies, which limit the reproducibility. In this respect, the adoption of common laboratory protocols, unique bioinformatics pipelines and shared analysis methods is fundamental to reduce external confounders [507]. In addition, it is known that differences in geography, diet and lifestyle can greatly influence the GM composition [91,508,509], and host genetics may play a major role [510]. Since many of these inherent covariates cannot be eliminated, the search for biomarkers and therapies focused on specific populations, as well as more “personalized” approaches, may be of interest and improve consistency [511,512]. However, the feasibility of these more targeted approaches highly depends on the availability of a large amount of data, which for the moment remains a limitation. When considering diagnosis, the multi-omics analysis of samples from PD patients at different stages of disease progression, including prodromal and early subjects is of utmost importance to better define stage-associated signatures [507]. Moreover, the lack of sufficient comparisons between microbial profiles obtained from patients under conventional pharmacological treatment versus treatment-naïve PD individuals limits their diagnostic applicability, since drugs intake may greatly affect GM composition [185,186,188]. Additionally, the dose and time of administration of GM-modifying drugs may also vary depending on the stage of the disease, thus possibly further influencing the study outcomes. Furthermore, the optimal probiotic-prebiotic cocktail to be employed has yet to be identified, as well as the best dietary intervention. Since a crucial aspect in the context of aging-related neurodegenerative diseases is prevention, forthcoming research should better assess if preventative benefits can be obtained by undergoing eubiosis-reestablishing therapies in a prodromal phase of the disease, rather than focusing entirely on disease treatment [351,513]. In the future, the administration of engineered microbes (also known as live biotherapeutic products), which carry the traits of interest, together with defined and balanced diets, may be an accessible, non-invasive and practicable intervention in support of pharmacological PD treatment, but more research is needed before a clinical application [215].

Overall, although much work remains to be done and large clinical trials are required, GM is emerging as a promising diagnostic and therapeutic tool for PD and deserves further investigation.

## Figures and Tables

**Table 1:** The effects of probiotics treatment regarding in vitro and in vivo experimental studies.

Probiotic	Experimental Model	Treatment Duration	Treatment Effects	Reference
*Lactobacillus salivarius*, *L. plantarum*, *L. acidophilus*, *L. rhamnosus*, *Bifidobacterium animalis* subsp. *lactis*, *B. breve*	In vitro. PBMCs from 40 PD patients	24 h	Increase of anti-inflammatory cytokines (IL-4, IL-10) Decrease of pro-inflammatory cytokines (TNF-α, IL-6, IL-17A) Reduction of ROS production	(Magistrelli et al., 2019) [328]
	In vitro. Caco-2 cells	2 h	Protection of epithelium from gut permeability	
	In vitro*. Escherichia coli* and *Klebsiella pneumoniae* inoculation	48 h	Inhibition of pathogen bacteria proliferation	
Symprove (*L. acidophilus*, *L. plantarum*, *L. rhamnosus*, *Enterococcus faecium*)	In vitro. Caco-2/THP1 cells	24 h	Improvement in epithelial tight-junction integrity, and in wound healing Increase of anti-inflammatory cytokines (IL-6, IL-10) Decrease of pro-inflammatory chemokine IL-8	(Ghyselinck et al., 2021) [329]
	In vitro. Stool samples from 3 PD patients	48 h	Modulation of GM composition (↑*Firmicutes*, ↓*Bacteroidetes*) Production of SCFAs and lactate	
*L. plantarum* 200655	In vitro. H_2_O_2_-treated SH-SY5Y cells	4 h	Increase of BDNF and TH mRNA expression Attenuation of apoptosis (↓apoptosis-related Bax/Bcl-2 ratio, ↓caspase-3 activity)	(Cheon et al., 2021) [330]
SLAB51 (*Streptococcus thermophilus*, *B. longum*, *B. breve*, *B. infantis*, *L. acidophilus*, *L*. *plantarum*, *L. paracasei*, *L. delbrueckii* subsp. *bulgaricus*, *L. brevis)*	In vitro. 6-OHDA-treated SH-SY5Y cells	2 h	Reduction in dopaminergic neuronal loss, by increasing the neuronal survival BDNF pathway (mBDNF, p-TrkB, p-ERK5, p-CREB, PI3K/Akt) and decreasing neuronal death pathway (pro-BDNF, p-JNK, p-ERK1,2, P75) Protection against OS (↓4-HNE).	(Castelli et al., 2020) [331]
	In vivo. 6-OHDA-treated mice	5 weeks	Improvement in motor impairment. Reduction in neuroinflammation (↓Iba1, ↓GFAP), and in OS, by restoring Nrf2/HO-1 activity and inhibiting NF-κB.	
*L. rhamnosus GG*, *B. animalis lactis*, and *L. acidophilus*	In vivo. MPTP- and rotenone-induced mouse model	4 weeks	Prevention of dopaminergic neurons loss by upregulation of BDNF and GDNF, and inhibition of MAO-B expression Amelioration of behavioural impairments. Raise in butyrate levels.	(Srivastav et al., 2019) [325]
*L. plantarum* PS128	In vivo. MPTP-induced mouse model	28 days	Alleviation of neuroinflammation (↓TNF-α, ↓IL-1β, ↓IL-6) and OS (↑SOD, ↑GSH, ↑CAT, ↑GPx). Improvement in motor deficits and dopaminergic neuronal cell death. Reduction of glial reactivity. Increase in BDNF expression and striatal dopamine level.	(Liao et al., 2020) [332]
*B. bifidum*, *B. longum*, *L. rhamnosis*, *L. rhamnosus* GG, *L. plantarum* LP28, and *Lactococcus lactis* subsp. *Lactis*	In vivo. MitoPark mouse model	16 weeks	Improvement in motor impairment, balance function, and motor coordination. Reduction in dopaminergic neuronal loss.	(Hsieh et al., 2020) [333]
*Clostridum butyricum*	In vivo. MPTP-induced mouse model	4 weeks	Reduction in motor impairment, dopaminergic neuronal loss (↑TH) and synaptic dysfunction (↑synapsin I). Inhibition of excessive microglia activation, via reversing GM dysbiosis. Increase in GLP-1 and GLP-1 receptor levels in the brain.	(J. Sun et al., 2021) [334]
*Lactobacillus plantarum DP189*	In vivo. MPTP-induced mouse model	14 days	Modulation of OS (↑SOD, ↑GSH-Px ↓MDA, ↓ROS), inflammation (↑IL-10, ↓TNF-α, ↓IL-6 and ↓IL-1β) and GM dysbiosis (↓*Proteobacteria*, ↓*Actinobacteria*, ↑*Lactobacillus*, ↑*Prevotella*). Reduction in α-SYN accumulation in the *substantia nigra*. Activation of Nrf2/ARE and PGC-1α pathways. Suppression of NLRP3 inflammasome.	(L. Wang et al., 2022) [335]
*Lactobacillus casei* Shirota	40 PD patients	5 weeks	Improvement in stool consistency. Reduction of bloating and abdominal pain.	(Cassani et al., 2011) [336]
*L. acidophilus* and *B. infantis*	40 PD patients	12 weeks	Reduction of bloating and abdominal pain.	(Georgescu et al., 2016) [337]
*L. casei*, *L. fermentum*, *L. acidophilus*, *B. bifidum*	50 PD patients	12 weeks	Reduction of IL-1, IL-8 and TNF-α gene expression. Increase in TGF-β and PPAR-γ.	(Borzabadi et al., 2018) [338]
*L. acidophilus*, *L. reuteri*, *L. fermentum*, and *Bifidobacterium bifidum*	60 PD patients	12 weeks	Reduction of UPDRS motor scores. Decrease of hs-CRP, MDA, insulin levels, and insulin resistance. Enhancement in GSH levels and insulin sensitivity.	(Tamtaji et al., 2019) [339]
Xexbio (*L. acidophilus*, *L. casei*, *L. lactis*, *B. infantis* and *B. longum*)	48 PD patients	8 weeks	Improvement of constipation and gut motility.	(Ibrahim et al., 2020) [340]
*L. acidophilus*, *L. reuteri*, *L. gasseri*, *L. rhamnosus*, *B. bifidum*, *B. longum*, *Enterococcus faecalis*, *E. faecium*	72 PD patients	4 weeks	Improvement of constipation, stool consistency and quality of life.	(Tan et al., 2020) [341]
Probio-M8 (*B. animalis* subsp. *lactis* Probio M-8)	82 PD patients	12 weeks	Amelioration in sleep quality, cognitive dysfunction, and defecation. Attenuation of anxiety, depression, and gastrointestinal symptoms. Modulation of gut microbiome, lipid metabolism, SCFAs and neurotransmitters serum levels.	(H. Sun et al., 2022) [342]

Abbreviations: 4-HNE: 4-hydroxynonenal; Bax: Bcl-2 associated X; Bcl-2: B-cell lymphoma 2; BDNF: brain-derived neurotrophic factor; CAT: catalase; GDNF: glial cell line-derived neurotrophic factor; GFAP: glial fibrillary acid protein; GLP-1: glucagon-like peptide 1; GM: gut microbiota; GPx: glutathione peroxidase; GSH: glutathione; GSH-Px: plasma glutathione peroxidase; hs-CRP: high-sensitivity C-reactive protein; Iba1: ionized calcium-binding adapter molecule 1; IL-1: interleukin 1; IL-10: interleukin 10; IL-17A: interleukin 17A; IL-1β: interleukin 1beta; IL-4: interleukin 4; IL-6: interleukin 6; IL-8: interleukin 8; MAO-B: monoamine oxidase-B; mBDNF: mature brain-derived neurotrophic factor; MDA: malondialdehyde; NF-κB: nuclear factor kappa-light-chain-enhancer of activated B cells; NLRP3: NLR family pyrin domain containing 3; Nrf2/ARE: nuclear factor erythroid 2-related factor 2/antioxidant response element; Nrf2/HO-1: Nrf2/hemoxygenase-1; OS: oxidative stress; PBMCs: peripheral blood mononuclear cells; p-CREB: phosphorylated cAMP response element-binding protein; PD: Parkinson’s disease; p-ERK1,2: phosphorylated extracellular signal-regulated kinase; p-ERK5: phosphorylated extracellular-signal-regulated kinase 5; PGC-1α: peroxisome proliferator-activated receptor-gamma coactivator-*1*alpha; PI3K/Akt: phosphoinositide 3-kinase/phosphorylated protein kinase B; p-JNK: phosphorylated c-Jun N-terminal kinase; PPAR-γ: peroxisome proliferator-activated receptor gamma; pro-BDNF: pro-brain-derived neurotrophic factor; p-TrkB: phosphorylated tyrosine receptor kinase B; ROS: reactive oxygen species; SCFAs: short chain fatty acids; SOD: superoxide dismutase; TGF-β: transforming growth factor beta; TH: tyrosine hydroxylase; TNF-α: tumor necrosis factor-alpha; UPDRS: unified Parkinson’s Disease rating scale; α-SYN: alpha-synuclein; ↑: increase; ↓: decrease.

**Table 2:** The effects of prebiotics treatment regarding in vivo experimental studies.

Prebiotic	Experimental Model	Treatment Duration	Treatment Effects	Reference
GOS, lcFOS, scFOS, nutriose	Rotenone-induced mice model	10 weeks	Improvement of motor symptoms, gastrointestinal dysfunction, and inflammation (↓GFAP, ↓T-cells infiltration). Restoration of DAT expression. Reduction of α-synuclein levels.	(Perez-Pardo et al., 2017) [372]
Polymannuronic acid	MPTP-induced model mice	5 weeks	Abolition of the apoptotic process (↓Bax, ↓Bax/Bcl-2 ratio). Prevention of dopaminergic neuronal loss (↑TH gene and protein expression in the striatum). Increase of faecal acetate, butyrate, and total SCFAs levels. Inhibition of striatal inflammation (↓TNF-α mRNA levels).	(Liu et al., 2022) [373]
Prebiotic residual medium obtained from *L. salivarius* subsp. *salicinium* AP-32 culture medium	6-OHDA-induced rat model	8 weeks	Reduction of dopaminergic neuronal loss, motor dysfunctions, and muscle atrophy. Increase of GPx and faecal SCFAs (propionate, butyrate). Restoration of mitochondrial function and energy metabolism.	(Nurrahma et al., 2021) [376]
Prebiotic residual medium obtained from *L. salivarius* subsp. *salicinium* AP-32 culture medium	6-OHDA-induced rat model	8 weeks	Amelioration of motor symptoms. Reduction of inflammation (↓TNF-α) and OS (↓ROS, ↑SOD, ↑GPx). Increase of SCFAs production (propionate, butyrate). Modulation of GM composition (↑*Ruminococcaceae*, ↑*Bifidobacterium*, ↑*Faecalibacterium*, ↓*Propionibacterium*, ↓*Clostridium*, ↓*Cylindriodes*, ↓*Ruminantium*).	(Tsao et al., 2021) [346]
Dietetic fiber supplements (wheat, pectin, dimethylpolyoxyhexane-900)	19 PD patients	8 weeks	Improvement in constipation and in motor function. Increase of total plasma levodopa levels.	(Astarloa et al., 1992) [374]
Psyllium	7 PD patients	8 weeks	Increase in stool frequency and weight.	(Ashraf et al., 1997) [375]
Resistant starch	57 PD patients	8 weeks	Improvement of non-motor symptoms. Reduction of calprotectin levels. Increase in butyrate concentration.	(Becker et al., 2021) [316]

Abbreviations: 6-OHDA: 6-hydroxydopamine; Bax: Bcl-2 associated X; Bcl-2: B-cell lymphoma 2; DAT: dopamine transporter; GFAP: glial fibrillary acid protein; GM: gut microbiota; GOS: galactooligosaccharides; GPx: glutathione peroxidase; lcFOS: long-chain fructooligosaccharide; MPTP: 1-methyl-4-phenyl-1,2,3,6-tetrahydropyridine; OS: oxidative stress; PD: Parkinson’s disease; ROS: reactive oxygen species; SCFAs: short chain fatty acids; scFOS: short-chain fructooligosaccharides; SOD: superoxide dismutase; TH: tyrosine hydroxylase; TNF-α: tumor necrosis factor-alpha; ↑: increase; ↓: decrease.

**Table 3:** The effects of dietary interventions in PD clinical trials.

Reference	Type of Study	Type of Dietary Intervention	Aim	Outcomes
Metcalfe-Roach et al., 2021 [383]	CrS	MIND or Medi	MIND/Medi vs. PD onset	Both diets delay PD onset; MIND slightly superior in the female subgroup.
Paknahad et al., 2020 [384]	CT	Medi	Medi vs. cognitive function	Improvement in executive function, language, attention, concentration, active memory and in the total score of cognitive assessment.
Rusch et al., 2021 [385]	CT	Medi	Medi vs. GI function	Correlation with weight loss, improved constipation, and modified gut microbiota in PD patients.
Cassani et al., 2017 [386]		Medi	Medi vs. PD progression	No significant correlation.
Maraki et al., 2019 [387]	CS	Medi	Medi vs. PD onset	Correlation with lower probability of prodromal PD in older people.
Zamzam Paknahad et al., 2022 [388]	CT	Medi	Medi vs. total antioxidant capacity (TAC) and PD severity	Improvements in TAC and PD severity.
Alcalay et al., 2012 [389]	CCS	Medi	Medi vs. PD status	Medi adherence is associated with PD age at onset.
Strikwerda et al., 2021 [390]	CS	Medi, Dutch diets	Medi, Dutch diets vs. PD risk	Protective effect.
Yin et al., 2021 [391]	CS	Medi	Medi vs. PD risk	Protective effect.
Agarwal et al., 2018 [392]	LS	MIND	MIND vs. PD development and progression	Decreased risk and slower progression of PD in older adults.
Lawrie et al., 2022 [393]	CrS	MIND	MIND vs. PD severity	Decreased fatigue and depression.
Koyuncu et al., 2021 [394]		KD	KD vs. PD patients voice quality	VHI * score improvement
VanItallie et al., 2005 [395]	Feasibility study	KD	KD vs. PD progression	UPDRS scores improvement.
Phillips et al., 2018 [396]	CT	KD vs a low-fat, high-carbohydrate diet	KD vs. PD progression	Motor and nonmotor symptoms improvement.
Tidman et al., 2022 [397]	CT	KD	KD vs. PD progression	UPDRS scores improvement.

Abbreviations: CCS: case-control study; CrS: cross-sectional study; CT: controlled trial; KD: ketogenic diet; LS: longitudinal study; Medi: Mediterranean diet; MIND: Mediterranean-DASH diet intervention for neurodegenerative delay; PUFAs: polyunsaturated fatty acids; UPDRS: Unified Parkinson’s Disease Rating Scale; VHI score: voice handicap index; * VHI is patient-rated scale developed to assess the level of disability experienced by patients affected by various voice disorders.

**Table 4:** Preclinical and clinical studies on the use of fecal microbiota transplantation for Parkinson’s disease.

Ref.	Study Cohort	Study Groups	Donor	Recipient	Experimental Procedure	Results	Adverse Events
Zhao et al., 2021 [470]	Mice	Controls (*n* = 15), rotenone (*n* = 15) and rotenone + FMT (*n* = 15)	Control mice	Rotenone-induced PD mice	Oral gavage (100 μL bacterial suspension) daily for 2 weeks	↓ Dysbiosis ↓ Motor symptoms ↑ Intestinal barrier and BBB integrity ↓ Systemic inflammation ↓ Neuroinflammation (SN) ↓ LPS (serum, colon and SN) ↓ TLR4/NF-κB pathway (colon and SN)	N.A.
Sun et al., 2018 [469]	Mice	Controls (*n* = 15), MPTP + PBS (*n* = 15) and MPTP + FMT (*n* = 15)	Control mice	MPTP-induced PD mice	Gavage (200 μL bacterial suspension containing 10^8^ CFU/mL) daily for 7 days	↓ Dysbiosis ↓ Fecal SCFAs ↓ Motor symptoms ↑ DA and 5-HT (striatum) ↓ Microglia and astrocyte activation (SN) ↓ TLR4/TNF-α pathway (gut and brain)	N.A.
Zhong et al., 2021 [471]	Mice	Controls + PBS (*n* = 10), controls + FMT (*n* = 10), MPTP + PBS (*n* = 10), MPTP + FMT (*n* = 10)	Control mice	Controls or MPTP-induced PD mice	Gavage (200 μL bacterial suspension containing 10^8^ CFU/mL) daily for 7 days	↓ Motor symptoms ↓ Fecal SCFAs ↓ α-syn (SN) ↓ Microglia activation (SN) ↓ TLR4/NF-κB pathway (striatum and SN)	N.A.
Zhang et al., 2021 [472]	Mice	Controls (*n* = 3), MPTP (*n* = 3) and MPTP + FMT (*n* = 3)	Control mice	MPTP-induced PD mice	Transplantation with 200 μL bacterial suspension (containing 10^8^ CFU/mL) daily for 2 weeks	↓ Neuroinflammation (SN) ↓ Motor symptoms ↑ *Blautia* ↓ *Anaerostipes*, *ASF356*, *Ruminococcus* and *Bifidobacterium* ↓ Microglia and astrocyte activation (SN) ↓ IL-1β, iNOS, GSK3β and p-PTEN (SN)	N.A.
Zhou et al., 2019 [473]	Mice	Mice pre-treated with MPTP and antibiotics, divided in PD-PBS (*n* = 8), PD-NA (*n* = 8), PD-NF (*n* = 8) and PD-NF/HK (*n* = 8)	Control mice or control mice undergoing FMT	MPTP-induced PD mice pre-treated with antibiotics	Gastric gavage (200 μL bacterial suspension containing 10^8^ CFU/mL) daily for 7 days	↑ DA and 5-HT (striatum) in PD-NF mice ↑ Neuroprotection in PD-NF mice	N.A.
Sampson et al., 2016 [40]	Mice	GF + FMT from SPF control mice, GF + FMT from PD patients, GF + FMT from healthy patients	Human PD patients (*n* = 6), human healthy controls (*n* = 6) or SPF control mice (*n* = 3)	α-syn-overexpressing mice	Oral gavage	In GF + PD-FMT mice: ↑ Physical impairment ↑ *Proteus*, *Bilophila* and *Roseburia* ↓ *Lachnospiraceae*, *Rikenellaceae*, *Peptostreptococcaceae* and *Butyricicoccus* ↓ Acetate ↑ Proprionate and butyrate	N.A.
Huang et al., 2019 [474]	Human (case report)	PD patient presenting tremor for 7 years and constipation (>3 years)	26 y.o. healthy male	71 y.o. male PD patients	Colonoscopy (200 mL of fecal microbiota suspension) daily for 3 days	↓ Tremor (no tremor for 2 months) ↓ Constipation ↑ α-diversity ↓ UPDRS score 1 week after FMT	No
Kuai et al., 2021 [475]	Humans (prospective single study)	PD patients	Frozen fecal microbiota from the China fmtBank	PD patients (*n* = 11)	Intra-intestine transplantation of 40–50 mL of frozen fecal microbiota resuspended in 200 mL saline solution	↑ *Blautia* and *Prevotella* ↓ *Bacteroidetes* ↓ H-Y, UPDRS and NMSS scores ↓ Wexner constipation and PAC-QOL scores	No
Xue et al., 2020 [476]	Humans	PD patients + FMT (via colonoscopy, *n* = 10; nasointestinally, *n* = 5)	5 Healthy donors (mean 22 y.o., 3 males and 2 females)	PD patients	Colonoscopy or nasointestinal administration	↓ PSQI, HAMA, PDQ-39, HAMD, UPDRS-III and NMSQ	5 cases: diarrhea (*n* = 2), abdominal pain (*n* = 2) and flatulence (*n* = 1)
Segal et al., 2021 [477]	Humans (uncontrolled case series)	6 PD patients with symptoms for 5 years (mean).	2 healthy donors (males, 38 and 50 y.o.)	6 PD patients with constipation (mean 52 y.o.), 3 males and 3 females	Colonoscopy (300 mL of fecal suspension)	↓ Motor and non-motor symptoms ↓ Constipation	1 case requiring hospitalization for observation

Abbreviations: α-syn: α-synuclein; BBB: blood-brain barrier; CFU: colony forming units; DA: dopamine; FMD: fasting mimicking diet; FMT: fecal microbiota transplantation; GF: germ-free;GSK3β: glycogen synthase kinase-3 beta; H-Y: Hoehn and Yahr scale; HAMA: Hamilton anxiety scale; HAMD: Hamilton depression rating scale; 5-HT: serotonin;IL-1β: interleukin 1 beta; iNOS: inducible nitric oxide synthase; LPS: lipopolysaccharide; MPTP: 1-methyl-4-phenyl-1,2,3,6-tetrahydropyridine; NF-κB: nuclear factor kappa-light-chain-enhancer of activated B cells; NMSQ: non-motor symptoms questionnaire; NMSS: non-motor symptoms scale; p-PTEN: phosphorylated PTEN; PAC-QOL: patient assessment of constipation quality of life questionnaire; PBS: phosphate buffered saline; PD-NA: MPTP-induced PD mice treated receiving FMT from control mice; PD-NF: MPTP-induced PD mice treated receiving FMT from control mice undergoing FMD; PD-NF/HK: MPTP-induced PD mice treated receiving heat inactivated FMT from control mice undergoing FMD; PD-PBS: MPTP-induced PD mice treated receiving PBS; PD: Parkinson’s disease; PDQ-39: Parkinson’s Disease Questionnaire; PSQI: Pittsburgh sleep quality index; SCFAs: short chain fatty acids; SN: substantia nigra; SPF: specific pathogen free; TLR4: toll like receptor 4; TNF-α: tumor necrosis factor α; UPDRS: unified Parkinson’s disease rating scale; y.o.: years old; ↓: decrease; ↑: increase.

## Data Availability

Not applicable.
